# Characterisation of Adaptive Genetic Diversity in Environmentally Contrasted Populations of *Eucalyptus camaldulensis* Dehnh. (River Red Gum)

**DOI:** 10.1371/journal.pone.0103515

**Published:** 2014-08-05

**Authors:** Shannon Dillon, Rachel McEvoy, Darren S. Baldwin, Gavin N. Rees, Yvonne Parsons, Simon Southerton

**Affiliations:** 1 CSIRO Agriculture Flagship, Canberra, ACT, Australia; 2 Department of Genetics, La Trobe University, Bundoora, VIC, Australia; 3 Murray Darling Freshwater Research Centre, Wodonga, VIC, Australia; 4 CSIRO Land and Water Flagship, Wodonga, VIC, Australia; Wuhan Botanical Garden, Chinese Academy of Sciences, Wuhan, China

## Abstract

As an increasing number of ecosystems face departures from long standing environmental conditions under climate change, our understanding of the capacity of species to adapt will become important for directing conservation and management of biodiversity. Insights into the potential for genetic adaptation might be gained by assessing genomic signatures of adaptation to historic or prevailing environmental conditions. The river red gum (*Eucalyptus camaldulensis* Dehnh.) is a widespread Australian eucalypt inhabiting riverine and floodplain habitats which spans strong environmental gradients. We investigated the effects of adaptation to environment on population level genetic diversity of *E. camaldulensis*, examining SNP variation in candidate gene loci sampled across 20 climatically diverse populations approximating the species natural distribution. Genetic differentiation among populations was high (F_ST_ = 17%), exceeding previous estimates based on neutral markers. Complementary statistical approaches identified 6 SNP loci in four genes (COMT, Dehydrin, ERECTA and PIP2) which, after accounting for demographic effects, exhibited higher than expected levels of genetic differentiation among populations and whose allelic variation was associated with local environment. While this study employs but a small proportion of available diversity in the eucalyptus genome, it draws our attention to the potential for application of wide spread eucalypt species to test adaptive hypotheses.

## Introduction

Trees are foundation species in many terrestrial ecosystems and changes to local environment associated with natural and anthropogenic climate change are projected to impact, or are already impacting, the health of forest tree populations and the ecosystems they service worldwide [1]–[5]. As a greater number of species confront significant environmental change, it is becoming important to understand the factors influencing the capacity of populations to adapt and to monitor these [6]–[8]. Forest populations commonly exhibit evolved mechanisms to cope with prevailing environmental conditions, evident from the characterisation of adaptive phenotypic and genetic diversity in environmentally contrasted populations [9]–[11]. However in order to persist populations must retain the ability to adapt when conditions change [12]. Broadly speaking, plant populations adapt to environmental change through a combination of mechanisms including reversible changes to their physiological or morphological phenotypes independent of genotype (e.g. phenotypic plasticity); adaptation of phenotypes via changes in allelic composition as a result of selection (e.g. genetic adaptation); and migration to more suitable environments (e.g. seed or pollen dispersal). In some forest trees these responses will be insufficient to track rapid climate redistribution [5], [13], depending on the rate and magnitide of enviroimnetal change, physiological tolerances, life-history strategies (e.g. generation time), dispersal abilities, population dynamics, interspecific competition and levels of genetic diversity [6], [13], [14].

Reversible plastic responses are important for short term adaptation of natural populations [15], but in the long term, permanent adaptations reflecting locally prescribed changes in the underlying genetics of adaptively important traits will be required to preserve population fitness. The rate at which genetic adaptation can occur, with respect to the rate of environmental change, will be key to the persistence of many species impacted by climate change [13], [16], [17]. The capacity for genetic adaptation depends on multiple factors including the level of pre-existing or standing genetic diversity, effective population size, strength of selection and life history traits such as generation time and fecundity [15], [18], [19]. Consequently rates of genetic adaptation are likely to be highly variable among species and populations.

Insights into a species potential for genetic adaptation could be gained through characterisation of the relative abundance of adaptive versus neutral variation in response to historic or prevailing environmental conditions, which might be applied to empirically test hypotheses about environmental adaptation. Forest trees are tractable models for adaptive genetic studies owing to widespread populations traversing environmental gradients, high levels of diversity and low frequency of co-segregation among gene loci (linkage disequilibrium) [10]. For tree species of commercial importance the availability of common gardens has revealed strong adaptive clines in wood, growth and phenology traits [9], [13], [20]. Population and landscape genetic studies have recently suggested abundant adaptive genetic variation underlying these differences [21]–[33]. Plausible links between adaptive genotypes and phenotypic variation give further insight into the biological basis for adaptive genetic variation in sevearl cases [10], [25].

Significant areas of forest and woodland growing in marginal or semi-arid regions of Australia are currently at risk of decline in response to climate change. The river red gum (*Eucalyptus camaldulensis* Dehnh.) is a large tree found in riparian zones and associated floodplains in arid and semi-arid regions throughout Australia [34]. River red gum depend on flooding for recruitment, but adults can withstand prolonged periods of drought. Changes to watering regime, resulting from decreased flood frequency as a result of both river regulation and prolonged drought (hotter, drier conditions) in south-eastern Australia, have caused populations of river red gum within the Murray-Darling Basin to decline [35]–[37]. The extent of genetic adaptation among widely distributed populations could inform our understanding of adaptation to climate in this species, as well as reveal candidate gene loci that may be important as genetic markers to assess adaptive potential and guide conservation.

Across the Australian continent *E. camaldulensis* traverses strong environmental gradients and has likely evolved mechanisms to cope with variation in water availability. Variation in morphology between provenances suggests populations may be locally adapted [38], [39]. Genetic diversity and evidence of genetic adaptation have also been assessed. A survey of genetic diversity based on microsatellite loci revealed high levels of genetic diversity that exhibits geographically defined structure [40]. Genetic differentiation among populations was correlated with environment, however this relationship was attributed to historical, demographic factors rather than selection. DNA sequence variation in *E. camaldulensis* revealed high levels of nucleotide diversity in genes within secondary metabolite biosynthetic pathways [41], and ratios of non-synonymous to synonymous polymorphism implied positive selection. Thumma et al. [42] performed whole transcriptome profiling to identify genes that may be important to drought response. Sequence analyses revealed high ratios of non-synonymous to synonymous polymorphism in nearly 300 genes which were identified as putative targets of positive selection, with a third of these being differentially expressed between drought treatments. A recent study of adaptive variation at the whole genome scale in *E. camaldulensis* sampled from four sites in northern Australia revealed signatures of adaptation based on nucleotide sequence level tests [33]. Nearly 2000 SNP loci were identified whose alleles were differentiated between pairs of environmentally contrasted sampling locations.

Given the broad geographic and environmental distribution of *E. camaldulensis* it is desirable survey patterns of diversity at genetic loci that could be targets of selection, e.g. coding genes, in population samples spanning the natural range of the species. The earlier microsatelite study extensively sampled the natural populations, however the marker system applied was not suitable for studies of adaptation. Conversely, studies of genetic adaptation have targeted only a narrow subset of the available population diversity or did not allow comparison among populations. In this study we examined genetic diversity and divergence of 59 SNP markers sampled from twelve candidate gene loci in 20 populations of *E. camaldulensis* distributed across the species natural range. Several tests for evidence of genetic adaptation were performed upon individual SNPs as well as set of SNPs representing whole genes, and correlations with environmental parameters were investigated via association studies. The results suggest selection has driven diversity among populations for some genes and highlights the amenability of this species for further landscape level studies of adaptation employing larger numbers of individuals, populations and SNP loci.

## Methods

### Populations

**Figure 1:**
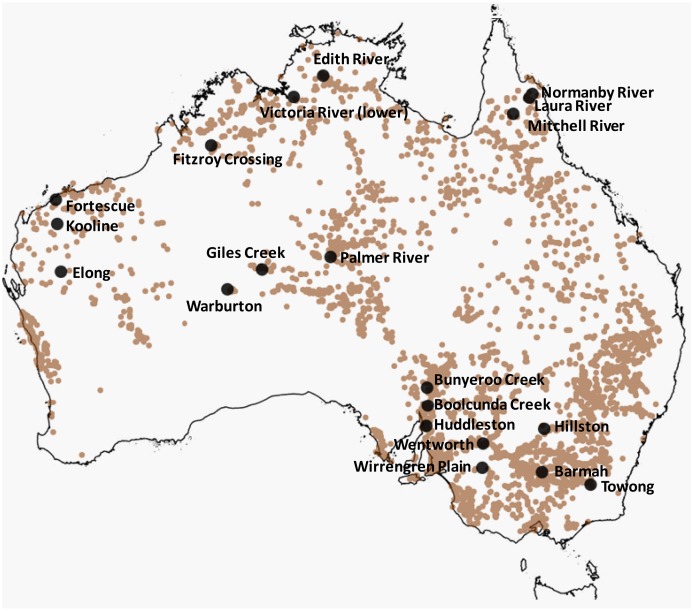
Location of *E. camaldulensis* populations sampled at 20 sites across mainland Australia. Occurrence records of *E. camaldulensis* downloaded from the Atlas of Living Australia (small circles) approximate the distribution of this species, which does not occur in Tasmania.

Ten trees were sampled per population across 20 populations spanning the natural distribution of *E. camaldulensis*, representing a subset of the collection previously published by Butcher et al. [40] (Table 1; Figure 1). Individual tree DNA samples were archived at the CSIRO Plant Industry laboratories, Canberra, and were used with permission. In total, 2 µg of diploid genomic DNA previously extracted from leaves using a modified CTAB protocol [43] was further purified on QIAGEN QIAquick PCR purification columns according to the manufacturer's instructions. Data for 15 microsatellite (SSR) loci previously generated by Butcher et al. [40] in the same 20 populations (with the exception of Wirrengren Plain) was made available and included in downstream analyses, serving as a benchmark for neutral, demographic effects on population genetic diversity.

**Table 1:** *E. camaldulensis* populations sampled at 20 sites across Australia.

population	region	state	taxon	no.	latitude	longitude	_CLIM_PCA 1	_CLIM_PCA2	_ECOL_PCA 1	_ECOL_PCA2	_GEOG_PCA1	_GEOG_PCA2
Barmah	Murray-Darling Basin	NSW	*Subsp. camaldulensis*	10	−35.50	145.07	−3.20	−0.27	1.35	0.13	−1.30	0.93
Boolcunda Creek	Spencer Gulf	SA	*Subsp. minima*	10	−32.18	138.28	−2.33	0.90	−0.65	0.21	0.60	1.34
Bunyeroo Creek	Spencer Gulf	SA	*Subsp. minima*	10	−31.24	138.25	−1.57	1.52	−0.66	0.53	−0.15	1.08
Edith River	Northern Australia	NT	*Subsp. obtusa*	10	−14.11	132.02	2.86	−3.14	1.67	0.92	−0.83	−1.19
Elong	Western Australia	WA	*Subsp. refulgens*	10	−25.15	116.41	1.66	2.04	−1.53	0.09	1.35	−1.85
Fitzroy Crossing	Northern Australia	WA	*Subsp. obtusa*	7	−18.11	125.36	3.20	0.54	0.01	0.02	−0.01	−1.89
Fortescue	Western Australia	WA	*Subsp. refulgens*	9	−21.18	116.09	2.87	1.14	−1.14	0.13	−0.94	−2.07
Giles Creek	Central Australia	WA	*Subsp. arida*	10	−25.04	128.40	0.95	2.67	−0.81	0.12	3.60	−0.25
Hillston	Murray-Darling Basin	NSW	*Subsp. camaldulensis*	10	−33.37	145.18	−2.16	0.36	−0.63	0.16	−1.73	1.67
Huddleston	Spencer Gulf	SA	*Subsp. minima*	5	−33.20	138.20	−3.04	−0.48	2.94	1.16	1.04	0.54
Kooline	Western Australia	WA	*Subsp. refulgens*	10	−22.55	116.17	2.49	1.91	−1.08	0.07	0.58	−0.59
Laura River	North Eastern Australia	QLD	*Subsp. simulata*	10	−15.39	144.31	1.42	−2.92	0.07	−0.46	−1.70	−0.99
Mitchell River	North Eastern Australia	QLD	*Subsp. simulata*	10	−16.31	143.38	1.99	−2.20	−0.51	−0.08	−1.41	0.14
Normanby River	North Eastern Australia	QLD	*Subsp. simulata*	10	−15.18	144.51	1.34	−3.29	1.01	−4.14	−0.86	−0.19
Palmer River	Central Australia	NT	*Subsp. arida*	10	−24.34	132.47	0.26	2.15	−0.87	−0.03	3.24	0.39
Towong	Murray-Darling Basin	NSW	*Subsp. camaldulensis*	10	−36.08	148.00	−4.21	−2.51	1.79	−0.03	−0.13	2.25
Victoria River (lower)	Northern Australia	NT	*Subsp. obtusa*	10	−15.37	130.28	2.44	−1.52	1.02	0.44	−2.24	−1.10
Warburton	Central Australia	WA	*Subsp. arida*	10	−26.09	126.33	1.07	2.67	−1.22	0.07	2.42	0.04
Wentworth	Murray-Darling Basin	NSW	*Subsp. camaldulensis*	10	−34.07	141.55	−2.62	0.51	−0.39	0.40	−0.99	1.39
Wirrengren Plain	Murray-Darling Basin	VIC	*Subsp. camaldulensis*	10	−35.26	141.53	−3.41	−0.07	−0.37	0.28	−0.53	0.33

no.  =  number of trees sampled per population.

Populations are a sub sample from the collection of Butcher *et al.* [40].

Subspecies nomenclature as per [95].

Principal component variables were calculated from three sets of environmental parameters relating to climate, ecology and geography.

### Environmental data

The populations sampled extend across the natural range of river red gum Australia wide, including six subspecies, traversing broad environmental gradients from central Australia to the wet tropics. Twenty one parameters reflecting variation in environment at each site were extracted from the Atlas of Living Australia website at http://www.ala.org.au, accessed 24 June 2013 (Table S1). To reduce redundancy in the data set the total number of environmental variables was reduced to a set of six multivariate traits following principle component analyses (PCA) implemented in the package StatistiXL (Table 1). PCA was performed separately on sets of variables grouped broadly into three environmental classes: geography, climate and ecology. Where more than one principal component was identified in each class, those with an Eigen value ≥1 and which brought the cumulative variance to ≥50% were selected. Component loadings describing the contribution of each environmental variable to the reduced component variables for each class are presented in Table S2. Each of the reduced environmental traits exhibited a strong relationship with latitude (Figure S1).

### Candidate genes, SNP selection and genotyping

Using single nucleotide polymorphisms (SNPs) we examined the variability of twelve genes in trees sampled across the natural range of *E. camaldulensis*. The candidate genes applied in this study were grouped into two functional categories: 1. plant water relations (PIP2, Dehydrin and ERECTA), and 2. xylem cell wall development (CAD, CCR, CESA1, CESA3, COBL4, COMT1, Korrigan, MYB4 and bZIP). The first group have the potential to impact plant water use in response to climate, via directing the movement of water between cellular compartments and out of the leaf stomata, or acting as internal cellular stabilisers in response to dehydration [44]–[46]. The second group moderate the physical properties of the vascular architecture and wood, which are shown to be important adaptive traits that can indirectly influence plant responses, for example drought [47]. Thus genetic diversity in both sets of genes could potentially reflect adaptation to environment.

Polymorphism data was identified from eleven candidate genes for which amplicons representing the open reading frame had previously been sequenced on a pooled sample of 500 *E. camaldulensis* individuals drawn from Murray-Darling Basin populations in the collection of Butcher et al. [40], using the 454 high throughput DNA sequencing platform (Roche) (unpublished data, Table 2). Sequences were trimmed and aligned against full length reference sequences obtained from the *Eucalyptus grandis* genome sequence (Phytozome: Eucalyptus grandis Genome Project 2010; http://www.phytozome.net/eucalyptus) in CLC Genomics Workbench (CLCbio). A twelfth gene, ERECTA, was sequenced in 36 individuals drawn from four Murray-Darling Basin populations of *E. camaldulensis* (Yanga National Park (12), Menindee (8), Wenthworth (8) and Wilcannia (8)), which were sampled from the collection of Butcher et al. [40] with the exception of Yanga which was sampled from a separate collection held at the CSIRO Plant Industry laboratories, Canberra. A 1000 base pair fragment of the ERECTA gene was amplified by PCR as above using primers designed from the coding regions. PCR products were sequenced by Macrogen Pty Ltd. Alignment and editing of the sequence data was performed using BioEdit [48].

**Table 2:** Candidate genes examined across *E. camaldulensis* populations.

abbreviated name	gene name	*E. grandis* homologue*	length**	putative function
CAD	Cinnamyl-Alcohol Dehydrogenase	Eucgr.G01350	900	lignin biosynthesis, [96]
CCR	Cinnamoyl-CoA Reductase	Eucgr.J03114	6000	lignin biosynthesis, [96]
CesA1	Cellulose Synthase 1	Eucgr.D00476	7000	cellulose biosynthesis, [97]
CesA3	Cellulose Synthase 3	Eucgr.C00246	7000	cellulose biosynthesis, [97]
COBL4	COBRA4 like gene	Eucgr.J01392	3000	cellulose biosynthesis, [98]
COMT	Caffeate 3-O-methyltransferase 1	Eucgr.A01397	2000	lignin biosynthesis, [96]
Dehydrin	Dehydrin like protein	Eucgr.I00186	1000	water stress response, [99]
ERECTA	Erecta leucine rich repeat protein	Eucgr.C0073	4500	water use efficiency, [45]
Korrigan	Korrigan (Endo-1,4-β-Glucanase)	Eucgr.G00035	2500	cell wall expansion, [100]
MYB4	MYB4 Transcription Factor	Eucgr.G03385	1800	lignin biosynthesiş [101]
bZIP	bZip Transcription Factor	Eucgr.F01867	9000	lignin biosynthesis, [102]
PIP2	Plasma Membrane Intrinsic Protein	Eucgr.D02548	3500	water stress response, [44]

*gene ID from annotated *E. grandis* genome sequence (www.phytozome.net).

**length of sequenced gene region in base pairs.

Single nucleotide polymorphisms were selected from DNA sequence alignments based on the following criteria: a base pair change was present, the base position was not an indel and the minor SNP allele occurred in at least three individuals. SNPs were selected to maximise coverage across the gene and avoid redundancy due to linkage disequilibrium, by selecting a SNP at least one to two hundred base pairs apart. Gene sequences were annotated, and for each SNP the base change, gene position (intron/exon) and amino acid substitution were recorded. Ten SNPs were chosen for each gene, with the exception of CAD (8), bZIP (7), Dehydrin (5) and Erecta (5). SNP genotyping was performed on genomic DNA samples using the Sequenom MassARRAY System at the Australian Research Genome Facility (AGRF) (Table S3). Genotype calls for all SNP loci across all individuals are provided in file Table S4.

Linkage disequilibrium (LD) in outcrossing forest tree species is typically low, and in eucalypts such as river red gum co-segregation of SNP loci along a gene decays within several hundred base pairs [41]. Consequently SNP markers typed in different genes might be expected to segregate independently. To test this, pairwise LD among genotyped loci was assessed using the program Tassel [49].

### Genetic diversity and divergence

In total, 76 of the 105 selected SNPs were successfully genotyped. Monomorphic SNPs and trees with >20% missing data were omitted from the data set, resulting in 59 SNPs typed across 191 individuals. Observed and expected heterozygosity and tests for Hardy-Weinberg Equilibrium (HWE) were performed on individual SNPs, and for sets of SNPs representing whole genes, for each population separately and overall using GenAlEx 6.2 [50].

Genetic divergence among populations based on all SNP loci was investigated using several complementary approaches. Firstly, analysis of molecular variance (AMOVA) was performed in GenAlEx to partition genetic diversity residing within and among populations and individuals. Using the AMOVA framework F_ST_ was also estimated in GenAlEx to obtain both overall and pairwise population estimates of genetic differentiation. Significance of the observed differentiation was tested by performing 1000 random permutations of the data. In the same way, F_ST_ was also estimated for these populations (with the exception of Wirrengren Plain) using polymorphism data for the 15 putatively neutral microsatellite loci (nuSSR) applied in Butcher *et al.* [40].

To compare patterns of genetic divergence inferred from the different marker systems correlations between matrices of pairwise population F_ST_ were examined via a Mantel test implemented in GenAlEx, and significance was based on 1000 permutations of the data. Divergence was also compared via principal coordinate analysis (PCoA) of genetic differentiation among populations implemented in GenAlEx. Spatial autocorrelation (isolation by distance or IBD) of population pairwise genetic divergence based on SNP loci was assessed using the Mantel function in GenAlEx, and significance was assessed on 1000 permutations. The overall pattern of genetic structure reflected in the SNP dataset was summarised as a reduced set of orthogonal axes following principal component analyses (PCA) in the package StatistiXL. The first 20 principal components, which had an Eigen value ≥1, and cumulatively accounted for ≥50% of the genotypic variance in the SNP data set, were used to describe genetic structure in association tests.

### Detection of adaptive genetic variation

In this study we investigate signatures of genetic adaptation in candidate genes that could underlie adaptive variation in environmentally contrasted populations of *E. camaldulensis*. Genetic structure in this species will potentially confound tests aimed to detect adaptation based on co-variation of genetic markers and environmental parameters, in light of the fact that neutral genetic variation and environment are autocorrelated among populations [40]. This feature arises from the tendency for genetically related populations to be geographically proximate (isolation by distance), and that proximate populations tend to share similar environments. Considering this we apply a conservative approach, relying on significance in multiple complementary tests, while accounting for neutral patterns of variation to infer adaptive signatures.

#### F_ST_ outlier tests

The SNP markers employed in this study reside within functional genes, and their allele frequencies may be subject to locus-specific effects such as selection. One approach to detect such effects is to assess observed differentiation (F_ST_) at individual loci (genes or SNPs) with respect to a neutral model [51]. Two methods (one Bayesian and one coalescent) were applied to test SNP marker differentiation against alternative neutral models.

Firstly, a neutral distribution of F_ST_ conditioned on heterozygosity (H_e_) ranging from 0 to 1 was generated via coalescent simulation over 40000 loci applying a Hierarchical Island Model that accounts for user defined population structure [52]. Genetic differentiation and hierarchical structure (defined by 100 demes and 5 nested groups) of the simulated data set was based on the 15 putatively neutral microsatelite loci (nuSSR) previously applied by Butcher et al. [40]. The distribution of simulated loci formed the basis of the neutral envelope against which F_ST_ for SNP loci were subsequently tested. The mean, 97.5% (upper) and 2.5% (lower) confidence intervals for the simulated F_ST_ distribution were calculated using ‘cplot’, which is distributed with the Fdist2 package [53]. Estimates of F_ST_ and H_e_ for 59 SNP loci were generated using ‘datacal’, also distributed with Fdist2. Estimates of F_ST_ and H_e_ averaged over whole genes were generated using GenAlEx. Significance of marker F_ST_ was tested using the ‘pv’ program distributed with Fdist2. Taking a conservative approach, markers which exceeded the upper or lower confidence intervals (P≤0.01) were classed as outlier loci. Additional outliers were also considered between 0.01<P<0.05. To account for multiple testing the false discovery rate (FDR) and q-values for each locus was estimated [54].

A second approach based on the multinomical-Dirichlet model was applied to identify SNP loci that may be under selection using the program BayeScan [55]. This method does not require prior knowledge of neutral population differentiation and in contrast to the island model can consider realistic ecological scenarios where size and migration rate differ among populations. Rather than comparing sites and testing for outliers, BayeScan estimates the probability of a locus being under selection for two models, one that includes the effect of selection and another that excludes selection, using a reversible jump Markov chain Monte-Carlo approach. The default parameters for the Markov chain were used (20 pilot runs of 5000 iterations, 100,000 total iterations), and the program was run twice to check reproducibility. Significance of SNP F_ST_ was interpreted from posterior odds using Jeffrey's scale [56].

#### Environmental associations

The spatial analysis method (SAM) of Joost et al. [57] was applied to detect individual SNP loci that may be locally adapted based on association with environmental variables. This test performed logistic regression of binary allele frequency data for all SNPs and environment on individual trees, where the significance of regression for each locus was tested based on p-values for two statistical tests a) likelihood ratio or G statistic, and b) the Wald statistic. Statistical significance in both tests was determined after applying the Bonferroni correction. The method aims to be conservative by calling an association based on significance in both tests. Allele scores for 59 SNPs scored across 191 trees were first converted to binary information (118 allele markers), where each allele was scored as a single locus. Component environmental variables, latitude and longitude for each individual tree were recorded in the same data frame.

Tests of association between allelic variation and environment were also performed while attempting to account for potentially confounding population genetic structure using two alternative approaches. Firstly associations between 6 component traits and 59 SNP loci were tested across 191 individuals via a least-squares fixed effect general linear model implemented in Tassel [49]. The statistical model is described by y  =  Xβ + e, where y is a vector for the observed dependent variable (environment), β is a vector containing independent fixed effects, including genetic marker and population structure matrices, X is the known design matrix, and e is the unobserved vector for the random residual (error) [58]. Significant divergence has been detected between populations of *E. camaldulensis* [40], consequently a matrix of individual scores for 20 principal components derived from the SNP data set describing genetic structure was incorporated in the model. P-values were corrected for experiment wise error following 1000 permutations of the data.

The second method was used to validate environmental associations at six outlier loci identified following analyses with both MatSAM and Tassel. This approach utilised a Bayesian framework to test correlations between allele frequencies at these loci and six component environmental variables against a null model specified by the covariance structure of allele frequencies across populations [59]. This was achieved by first generating a covariance matrix of allele frequencies among populations via a Monte Carlo Markov chain. Putatively neutral makers should be used for this step, thus we applied the entire SNP data set excluding the six putatively adaptive loci presented in Table 3. Default parameters for the Markov chain were used (20 pilot runs of 5000 iterations, 100,000 total iterations). The posterior of the covariance matrix was then applied as the null model to investigate whether allele frequencies for our loci of interest are correlated with environment using a Bayesian framework. Evidence for correlations between environment and SNP loci was interpreted based on Jeffrey's scale [56].

**Table 3:** Significance in multiple tests for selection suggest diversity at SNP loci from four genes may reflect local adaptation.

				Outlier tests				Association tests						Partial Mantel	
locus	gene	SNP	type	F_ST_^Fdist^	p-val^Fidst^	F_ST_^BayeScan^	Log(odds)^BayeScan^	ENV	p-val^Wald^	p-val^LRT^	p-val^GLM^	R^2 GLM^	Log(odds)^MCMC^	R^2 Mantel^	p-val^Mantel^
SNP29	COMT	A/G	silent	0.4	2.80E–03	0.21	ns	_ECOL_PCA1	8.83E–07	1.40E–07	0.001	0.122	0.51	0.027	0.102
								_CLIM_PCA2	8.35E–06	8.07E–06	0.002	0.074	0.70	0.005	0.218
SNP32	Dehydrin	A/G	synonymous	0.34	6.80E–03	0.26	ns	_CLIM_PCA1	9.76E–05	1.08E–04	0.001	0.061	0.62	0.041	0.009
								_GEOG_PCA2	6.35E–06	4.24E–06	0.001	0.088	ns	0.102	0.002
SNP33	Dehydrin	G/T	synonymous	0.40	2.10E–03	0.30	ns	_GEOG_PCA2	ns	ns	0.017	0.043	0.54	0.123	0.001*
SNP37	ERECTA	G/T	silent	0.45	2.00E–05	0.42	1.74	_CLIM_PCA1	6.68E–11	5.55E–15	0.024	0.033	ns	0.192	0.0001*
								_GEOG_PCA2	ns	ns	0.054	0.039	ns	0.102	0.001*
SNP56	PIP2	C/T	synonymous	0.34	6.20E–03	0.31	0.54	_CLIM_PCA1	1.67E–09	6.66E–16	ns	ns	ns	0.292	0.0001*
								_GEOG_PCA2	ns	ns	ns	ns	ns	0.096	0.001*
SNP58	PIP2	C/T	silent	0.45	8.30E–04	0.37	1.78	_CLIM_PCA1	2.19E–11	3.33E–16	0.014	0.041	0.91	0.102	0.001*

F_ST_: Wright's fixation index estimated in Fdist or Bayescan.

p-val^ Fdist^: significance of outlier test with hierarchical structure.

ENV: component variables derived from environmental data.

p-value^Wald^  =  significance of Wald test in SAM.

p-value^ LRT^  =  significance of likelihood ratio test in SAM.

Log(odds): logarithm (base 10) of Posterior Odds for alternative model in BayeScan and the MCMC method of Coop et al. 2010.

p-val^GLM^: significance of association test with environment in Tassel.

R^2 GLM^: proportion of environmental variance explained by SNP in Tassel.

R^2 Mantel^: correlation between environment and SNP F_ST_ matrices.

p-val^Mantel^: significance of Mantel correlation.

*Significance level (α) for partial Mantel test while performing Bonferoni correction was 0.001.

#### Isolation by adaptation

Genetic relationships estimated upon loci that have been targets of local adaptation might be expected to reflect differences in local selection pressure between populations [60]. Tests for covariance of pairwise matrices capturing genetic and environmental dissimilarity, such as the Mantel test, can be applied to examine relationships between environment and population genetic differentiation (isolation by environment) to identify potentially adaptive structure. However in performing such tests there is a need to isolate the effects of environment from those of population history on genetic differentiation, by incorporating information on patterns of divergence estimated from known neutral markers (i.e. nuSSR).

Partial Mantel tests were subsequently performed in the R package Ecodist [61], to test for covariance between pairwise population genetic differentiation (F_ST_) at SNP loci identified as possible targets of diversifying selection (Table 3) and pairwise dissimilarity for component environmental variables (Table 1). Pairwise population F_ST_ was estimated independently for the six SNP loci listed in Table 3 using Alrequin ver. 3.5 [62]. Prior to performing tests, linearity of the relationship between matrices was confirmed by viewing correlograms generated using the “pmgram” function (divided into 12 genetic distance bins) following Goslee et al. [61]. Using the “distance” function, component environmental parameters were converted into a matrix of population pairwise environmental Euclidean distances. Correlations between matrices of genetic differentiation for each of the putatively adaptive SNP loci and dissimilarity for each of six component environmental variables were examined using the “mantel” function. This was performed both with and without partialling out demographic effects on genetic differentiation by including a third matrix of pairwise population genetic differentiation estimated from 15 nuSSR loci. Significance was tested with 1000 permutations of the data in each case.

## Results

### Genetic diversity and divergence

SNP diversity generally conformed to Hardy Weinberg expectations within populations (55 out of 59 loci). When analysed as a combined sample, 76 percent of SNPs departed expectations, reflecting a Wahlund effect due to population structure. When estimated over all SNP loci, genetic diversity was moderate (H_e_ = 0.22) ranging from 0.19 at Normanby River (QLD) to 0.26 at Fortescue (WA). The overall level of LD between SNPs was low with only 0.6% of pairwise correlations between sites (R^2^) exceeding 0.2. This indicates that the majority of loci screened segregated independently across the 191 individuals sampled. Genetic differentiation (F_ST_) estimated on all SNPs among populations (F_ST_ = 0.17, P<0.01) was greater than the estimate based on 15 nuSSR loci in the same populations (excluding Wirrengren plain) (F_ST_ = 0.08) [40]. Further breakdown of genetic variation via analysis of molecular variance (AMOVA) revealed that 60% of the genetic variance among populations based on SNP loci occurred within individuals, while 23% was attributed to variation among individuals, and 17% among populations.

Pairwise population divergence estimated from SNP and SSR loci were moderately correlated (R^2^ = 0.27, p<0.001) (Figure S2), suggesting that while overall divergence was higher among SNP loci, the hierarchical relationships among populations were similar. Genetic relationships among populations, inferred from principal coordinate analysis (PCoA), showed clear geographic trends (grouping of populations by subspecies) and also point to similarity between SNP and SSR divergence at the level of subspecies (Figure S3). Differences were also evident, for example, populations belonging to the subspecies *arida* (central Australia) and *refulgens* (pilbara) had greater affinity to subspecies *minima* when relationships were inferred from SNP markers than nuSSRs. Isolation by distance (IBD) was found to be significant when divergence at SNP loci among population pairs was compared with physical distance separating populations (R^2^ = 0.36, p<0.001) (Figure S4). This result suggests that the distance over which pollen and seed are dispersed is likely to be a major determinant of gene flow in this species. Significant IBD was also identified by Butcher et al. [40] among populations of *E. camaldulensis* based on SSR loci.

### Detection of adaptive genetic variation

#### F_ST_ outlier tests

**Figure 2:**
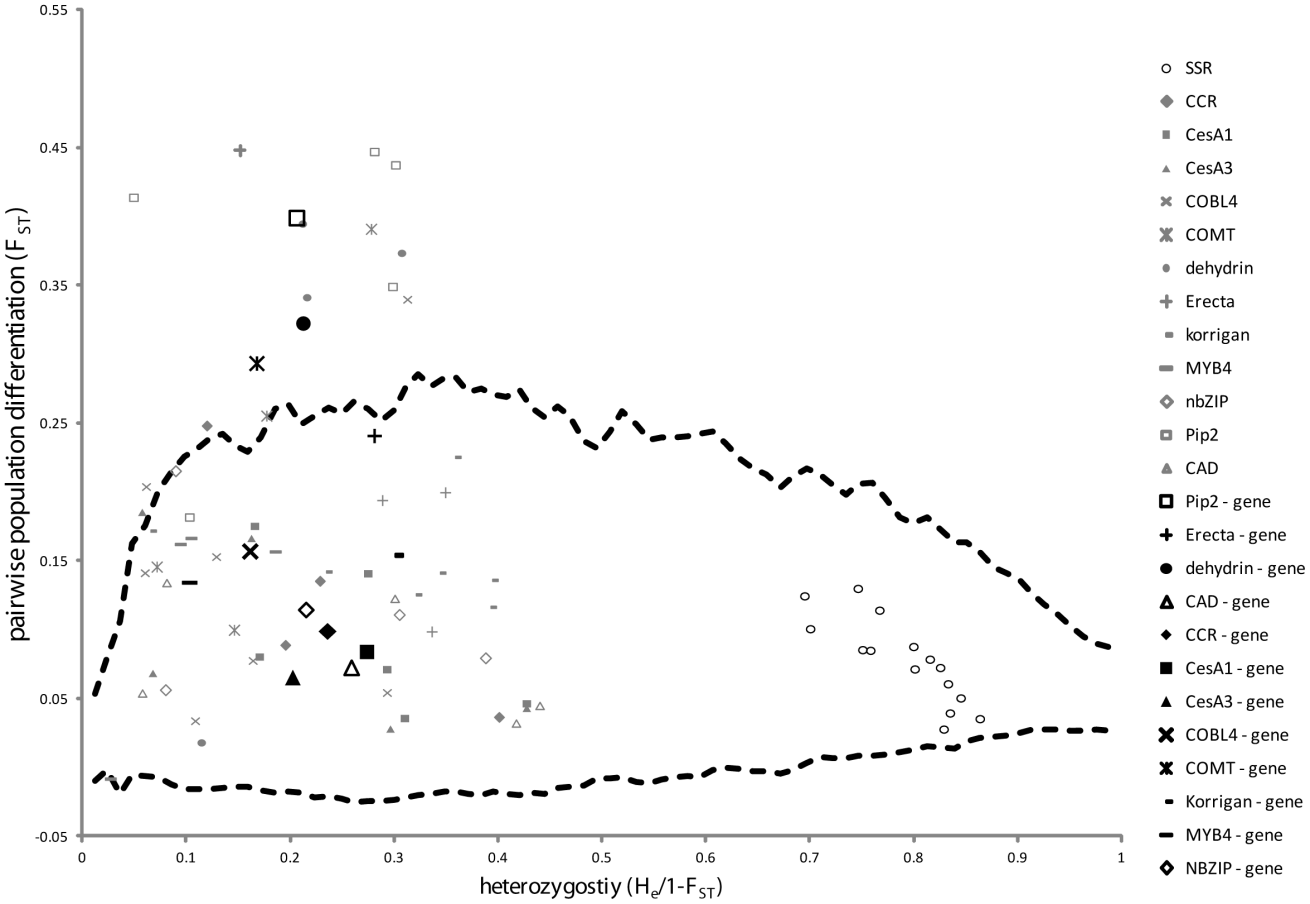
F_ST_ estimates for individual SNP loci and whole genes plotted as a function of heterozygosity. F_ST_ estimates were also plotted for the set of 15 SSR markers applied in Bucher *et al.* 2009. F_ST_ estimates for SNPs or genes that sat above the upper or lower boundary of the neutral envelope simulated in Arlequin were considered as potential targets of diversifying or homogenising selection respectively.

To examine whether variation in SNP allelic diversity among populations could be explained by selection, differentiation (F_ST_) at candidate loci (whole genes and SNPs) was compared to a neutral F_ST_ distribution simulated under a hierarchical island model [52]. F_ST_ outlier tests identified ten SNPs (from: PIP2 (4 SNPs), Dehydrin (3 SNPs), Erecta (1 SNP), COBL4 (1 SNP) and COMT1 (1 SNP)), as well as three genes (PIP2, Dehydrin and COMT1), whose divergence was greater than expected under neutrality (p<0.01), and whose pattern of allelic diversity among populations might be accounted for by diversifying selection (Figure 2; Table S5). We also identified three SNPs and one gene (ERECTA) which were significant at the less conservative threshold of p<0.05. Outlier loci accounted for 22% of all tests performed, exceeding the experiment wise error rate (α) of 5%, suggesting a low false positive rate overall. This is supported by significance of the FDR statistic, or q-value, for outlier loci which ranged between 0 and 0.05 (Table S5).

Outlier testing using an alternative Bayesian analyses identified three loci (from: Erecta (1 SNP) and PIP2 (2 SNPs)) where evidence in support of the model including diversifying selection was substantial (Log_10_Bayes factor >0.5<1) to very strong (Log_10_Bayes factor >1<2) (Table S6). Two additional loci from Dehydrin showed weak evidence for the adaptive model (Log_10_Bayes factor >0<0.5) based on Jeffrey's scale. Importantly, each of these loci were also identified by the first method employing coalescent simulations. BayeScan is expected to be a more conservative test for outliers than other methods, as it allows for deviations from a simple island model. Model averaging from the posterior distribution in BayeScan also tended to underestimate SNP differentiation (F_ST_) compared to the method-of-moment estimates from allele frequency variance components in Fdist2 and GenAlEx.

**Figure 3:**
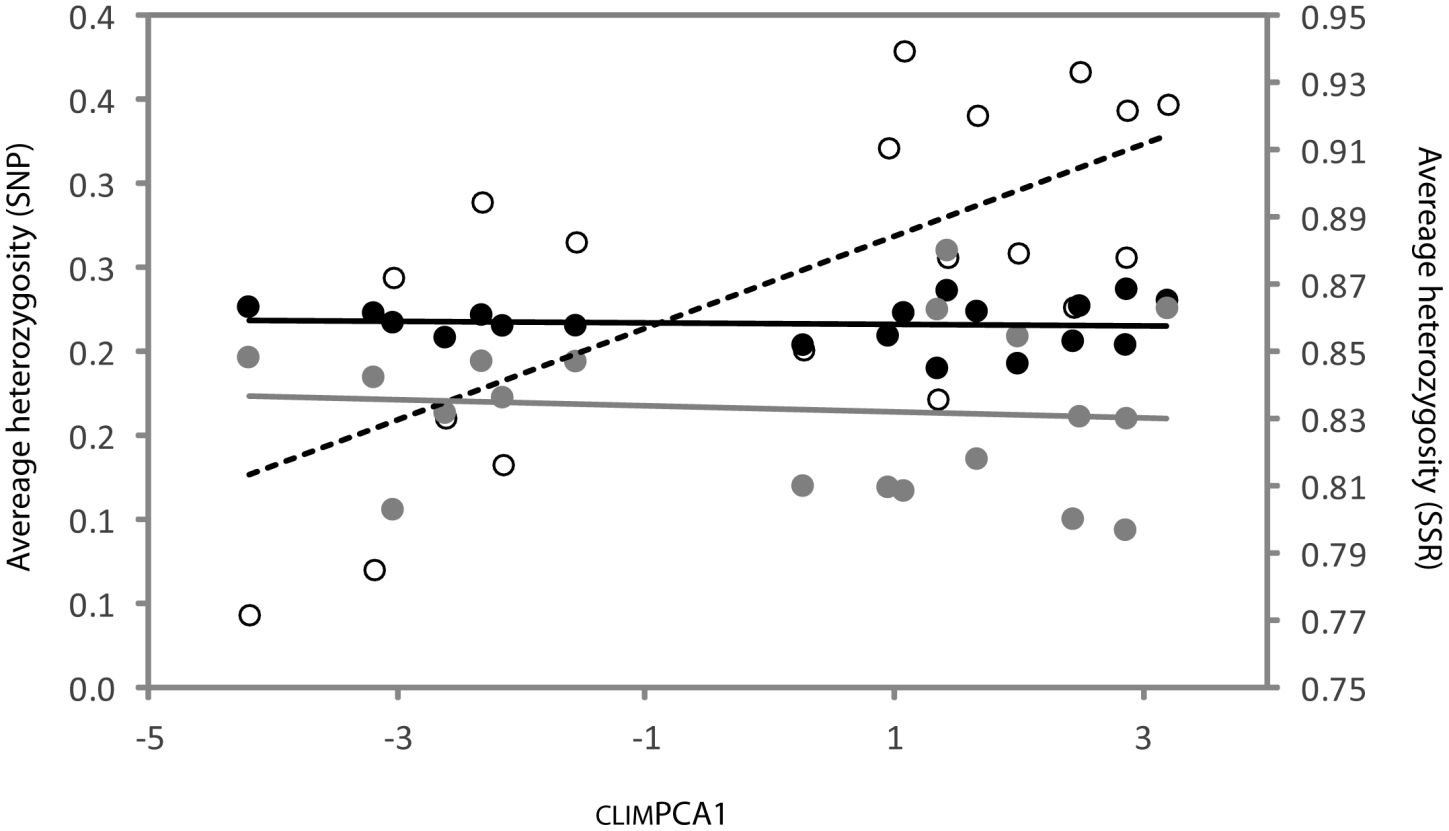
Covariance between average heterozygosity and environment (_CLIM_PCA1) among populations for 10 outlier SNP loci (white circles: R^2^ = 0.47; p<0.001) 49 non-outlier SNP loci (black circles: R^2^ = 0.002; p<0.85) and 15 nuSSR markers (grey circles: R^2^ = 0.008; p<0.72). Average heterozygosity for SNP and nuSSR markers are plotted against the left and right vertical axes respectively.

The adaptive hypothesis suggested for several SNP loci following outlier tests was further supported by covariance of heterozygosity (H_e_) and environment among populations. Population level heterozygosity estimated from a reduced data set containing outlier SNP loci only was significantly correlated with environment (e.g. _CLIM_PCA1 (R^2^ = 0.48, p<0.001), ecolPCA1 (R^2^ = 0.30, p<0.015) and geogPCA2 (R^2^ = 0.45, p<0.002)) (Figure 3). Covariance of H_e_ and environment was also observed at the level of individual outlier genes, where average H_e_ for PIP2, Dehydrin and COMT was positively correlated with _CLIM_PCA1 (Figure S5).

#### Association of allelic and environmental variation

To investigate further the adaptive evolutionary model suggested for some SNP loci following outlier tests, we looked for evidence of variation in selective constraints among populations that could explain allelic diversity. Tests of association between allelic variation and component environmental variables were performed using several alternative approaches. Firstly, logistic regression of allelic variation and component environmental variables was performed using MatSAM. This revealed associations between environment and allele frequency that were significant for both the LRT and Wald test following Bonferoni correction for number of loci, and supported a hypothesis of adaptive evolution at eleven outlier loci (Table S7). However the method implemented in MatSAM does not account for the presence of population structure, previously detected among river red gum populations, and may be prone to identifying false positive associations [63].

To correct for population structure SNP-environment associations were tested via two further approaches, a general linear model (GLM) implemented in the program Tassel to test for covariance of genotype and environmental scores across individuals, [49], and a Bayesian model to test for covariance of allele frequencies and environment across populations [59]. Population structure was accounted for in the GLM and Bayesian models as either a matrix of component environmental scores, or a SNP covariance matrix estimated via a MCM chain respectively. In total, six SNP loci previously identified as F_ST_ outliers were significantly associated with one or more environmental variable under the GLM (Table 3, Table S8 and Figure S6). The inclusion of population structure in this model reduced the number of associations by 70%, suggesting that without correction the false positive rate due to neutral structure is high. Estimated SNP effect sizes (R^2^ or the proportion of environmental variance explained by the SNP locus), ranged between three and twelve percent, which at the upper end is large compared to those typically observed for quantitative traits in trees [64]–[66]. Inflated effect sizes for some loci could reflect increased variance in environmental values due to the underpowered nature of the study owing to the small number of populations and individuals sampled [67].

**Figure 4:**
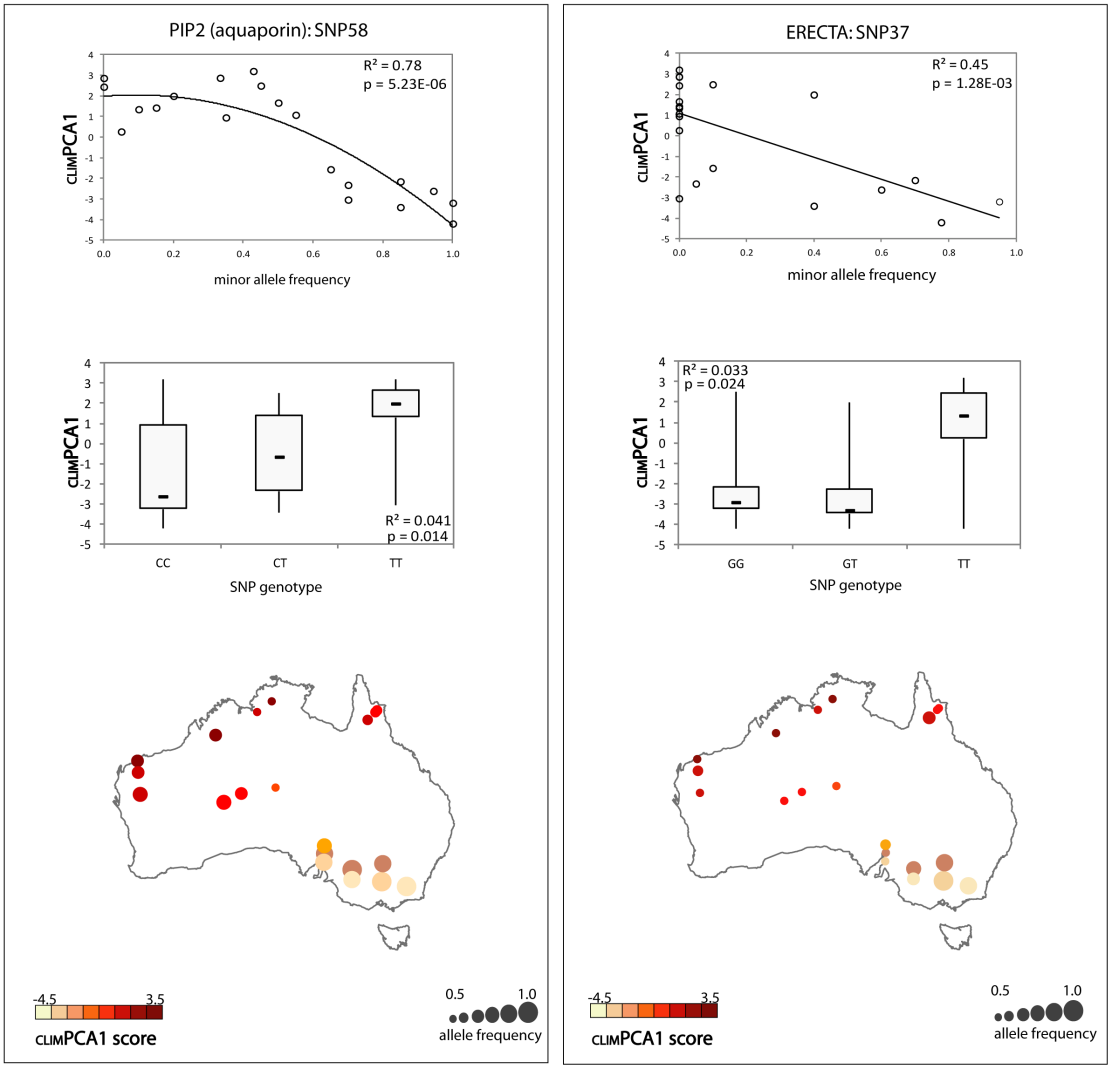
Minor allele frequency plotted as a function of environment (_CLIM_PCA1) for two outlier loci (top), for SNP 58 (C allele plotted, C/T SNP) (PIP2) and SNP 37 (G allele plotted, G/T SNP) (ERECTA); (middle) significant genotypic associations are illustrated as box plots for _CLIM_PCA1 as a function of genotype, and (bottom) in each case the map illustrates spatial and environmental structuring of allelic diversity, potentially reflecting local adaptation at these loci. The R^2^ and p-values displayed on each plot represent the proportion of variance in environmental parameters explained by the SNP maker and significance of the observed relationship.

The method of Coop was more conservative, possibly owing to the small size of the SNP data set and potential for non-neutral loci among SNPs applied in estimating the covariance matrix. Evidence for correlations between environment and four of six SNP loci tested using the Bayesian framework was substantial (Log_10_Bayes factor >0.5<1) based on Jeffrey's scale (Table 3). The associations summarised in Table 3, those presenting strong evidence for local adaptation in multiple tests, are illustrated as a function of environmental variation in Figures 4 and S6.

#### Genetic structure at adaptive loci

**Figure 5:**
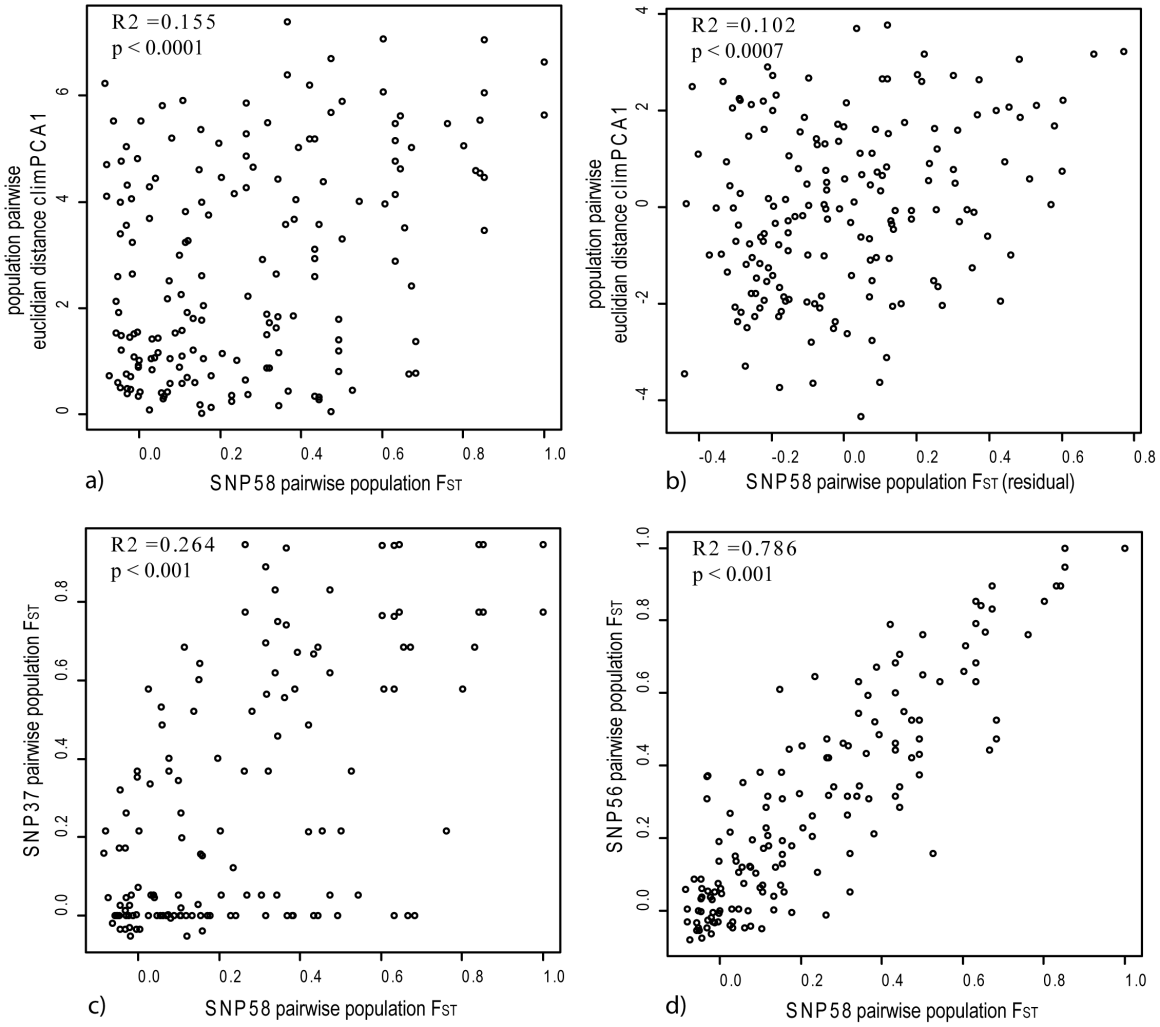
Covariance of pairwise genetic differentiation (F_ST_) for SNP58 (PIP2 gene) and environment may be indicative of adaptive genetic structure, as suggested by pairwise population F_ST_ for SNP 58 visualised as a function of (a pairwise population dissimilarity for climPCA1, and b) pairwise population dissimilarity for climPCA1 adjusted for demographic effects on genetic diversity, where both variables are plotted as the residuals of their linear model with population pairwise F_ST_ based on 15 putatively neutral SSR loci [40]. Population pairwise F_ST_ for SNP58 exhibited significant covariance with estimates for two putatively adaptive loci c) SNP37 (ERECTA), and d) SNP56 (PIP2). Correlation coefficients (R squared) and p values for Mantel (a, c, d) and partial Mantel (b) tests are presented in each case.

The relationship between environmental dissimilarity and hierarchical population structure inferred from putatively adaptive loci was assessed via a partial mantel test, while accounting for demographic signals inferred from SSR markers. This approach identified several outlier SNP loci, where an allelic relationship with environment had been detected, that exhibited genetic structure reflecting environmental differences among populations (or isolation by environment) (Table 3, Figure 5). In total, dissimilarity matrices for five SNP-environment combinations were significant following Bonferoni correction (p≤0.001). The results suggest that the selected environments could have driven genetic structure in these cases, and supports inferences of adaptive selection at these loci from outlier and association tests. Pairwise divergence at SNP58 from the Pip2 gene is illustrated as a function of dissimilarity in _CLIM_PCA1 which was significant both before and after accounting for neutral structure (Figure 5). We also observed that differentiation at this locus was significantly correlated with genetic differentiation for SNP37 (ERECTA) and SNP56 (PIP2) (p≤0.001), which were similarly correlated with dissimilarity for _CLIM_PCA1 (Table 3).

## Discussion

Natural populations occurring across environmental gradients offer opportunities to detect signatures of selection resulting from local adaptation, and test specific hypotheses about the spatial, environmental and temporal scales over which adaptive evolution is exerted [10], [11], [51]. In trees, studies of adaptive evolution have primarily focused on adaptation to long-standing environmental clines because of their long generation times [10]. Here we examined genetic diversity and evidence of adaptation at candidate gene SNP loci sampled in environmentally contrasted natural populations of *Eucalyptus camaldulensis* across the species range. These populations are differentiated for climate, primarily reflecting variation in rainfall, evaporation, temperature and sunlight which potentially drive differences in water availability, a strong driver of adaptation in diverse plant species [68], [69]. The populations were also variable for a number of ecological indices, soil and geographic features which could act as selective constraints [70], [71]. Throughout the early to mid Holocene conditions over most of Australia were wetter than the present day, and climate records suggest the onset of modern conditions from around 4000 years ago [72], [73]. We therefore expect that differences in climate among populations inferred from modern instrumental recordings have prevailed sufficiently long to provide opportunities for local adaptation over multiple generations.

Local adaptation among provenances and populations of *E. camaldulensis* has previously been suggested from variation in adaptive phenotypes which correspond to local environment including: morphological traits (growth form, leaf thickness, stomatal density, depth of root system, root to shoot ratio and phenology) [38], [74]–[77]; growth rate (height, diameter) [78]; wood properties (density, shrinkage, fibre length) [79], [80]; physiological responses (water use efficiency, stomatal conductance, CO_2_ assimilation) [81]–[83]; and drought tollerance [76], [81], [82], [84], [85]. The adaptive clines suggested from phenotypic variation are supported by recent evidence of genetic adaptation within coding genes [33], [41], [42]. In the present study we surveyed a modest number of candidate gene SNP loci in a broad sample of environmentally contrasted *E. camaldulensis* provenances approximating the species natural distribution. This approach provided for the first time a coarse indication of the distribution of potentially adaptive variation in this species at a broad landscape scale.

### Adaptive variation in candidate genes

We identified six putative examples of adaptive evolution based on variation in SNP allele frequencies among populations and co-variation with environment (Table 3). These SNP loci, located in four genes, were significant in outlier tests and also were significantly associated with environment in MatSAM and one or both of Tassel and the MCMC method of Coop et al. [59]. Additionally, partial mantel tests revealed several loci where population genetic relationships were shown to be correlated with environmental dissimilarity among populations, suggestive of local adaptation. Of the six SNP loci identified as possible targets of diversifying selection, the majority (83%) reside in genes predicted to have direct roles in plant water relations (e.g. PIP2, Dehydrin and ERECTA). The over representation of outlier loci from this functional class is perhaps compelling given SNPs within “water use” genes accounted for only 25% of the data set. This suggests that genes directly impacting water movement or dehydration response for example may be more likely to be subject to adaptation in populations where climatically driven water availability is contrasted compared to genes with other functions, such as structural cell wall genes. This is consistent with studies identifying aquaporins and dehydrins as candidates for adaptive evolution in response to water availability [22], [28], [86]–[88]. Erecta has previously been shown to regulate transpiration efficiency in *Arabidopsis* [45], and has been linked with drought adaptation in provenances of *Populus nigra* (Viger, unpublished data). In a recent gene expression study in *E. camaldulensis* both PIP2 and ERECTA were found to be differentially expressed (down regulated) in droughted compared to well watered *E. camaldulensis* plants [42]. This points to the importance of two of these genes in response to drought and as possible targets of selection for adaptation in natural populations.

### Environmental associations

Associations with environment provided support for local adaptation to explain allele frequency variation among populations at outlier loci. Significant co-variation of average expected heterozygosity (H_e_) for outliers and climate initially suggested this (Figure 3; Figure S5). The tendency for higher diversity at hotter, drier sites could potentially reflect a clinal shift in balancing selection favouring heterosis [89], [90], however this explanation is not supported by observed numbers of heterozygotes, and the trend is more likely to coincide with directional selection of alleles along the environmental cline. Significant association of allele frequency and genotype with environment, mainly climate (_CLIM_PCA1), was subsequently confirmed for several outlier loci using three different methods while accounting for demographic effects (Table 3). The results suggest that variation in climate, specifically temperature and evaporation is potentially important as a driver of adaptation in this gene set, and to a lesser extent rainfall, species richness and soil type.

The best supported cases for selection are illustrated by two outlier SNP loci, namely SNP37 (G/T) from Erecta (a putative leucine-rich repeat receptor-like kinase) and SNP58 (C/T) from PIP2 (an aquaporin), which were both associated with climate (_CLIM_PCA1) and exhibited significant adaptive structure (Figure 4; Table 3). These loci exhibit similar clines in allele frequency with respect to _CLIM_PCA1 which could indicate positive directional selection. Low levels of linkage disequilibrium (LD) in natural populations of river red gum [41] could mean that selection may be acting on these loci directly, or on a closely linked locus. The frequency of the minor (C) allele of SNP58 is decreased in populations where both temperature and evaporation is highest (e.g. more positive values of _CLIM_PCA1). Tests of association indicate this cline in allele frequency reflects a greater proportion of T:T homozygotes at this locus in the driest populations. The heterozygote at this locus is associated with climates intermediate to the two homozygotes, suggesting an additive mode of gene action on an unknown adaptive trait. Similarly for ERECTA, an increase in the minor (G) allele frequency was associated with reduced values of _CLIM_PCA1 (wetter, more mesic, conditions). The cline in allele frequency at this locus reflects a greater proportion of T:T homozygotes in the driest populations, and both the heterozygote G:T and homozygote G:G are associated with lower temperature and evaporation, suggesting a dominant mode of gene action (Figure 4). Both of these loci are silent, occurring in intronic sequence and do not code a change in the predicted protein product. It is possible that the target of selection is a closely linked locus which is amino acid changing, however it is also feasible that these silent mutations could be functional variants which are themselves under selection. Examples of functionality of non-coding polymorphisms, including cis-acting regulatory elements, have been observed in eucalypts and other species [91], [92].

The relative grouping of populations based on allele frequency and environment for the two loci in Figure 4 suggests relationships that are congruent with geographic and neutral population structure. The eight populations with low values of _CLIM_PCA1 and high frequency of the C allele for SNP58 belong to either the Murray-Darling Basin or Spencer Gulf provenances. A similar pattern is observed for SNP37. While it is possible that autocorrelation of climate and population demography in this species [40] has increased the risk of false association, we have been careful to account for neutral population structure in these analyses. The bias towards water use genes among associated SNPs also suggests a functional basis to the co-variation with environment. These results implore the use of multiple complementary approaches and careful consideration of potentially confounding population structure in studies aiming to differentiate between allelic variation arising from adaptation and neutral demographic processes in this species.

Genetic divergence (F_ST_) among populations for several putatively adaptive loci was related to population level dissimilarity for _CLIM_PCA1 and _GEOG_PCA2 (Table 3). The results suggest that the specific environments loading to each multivariate parameter could have constrained genetic relationships among populations at these loci, and provide support for inference of local adaptation from outlier and association tests. Similarities in the inferred genetic relationships were observed for some loci. For example, pairwise population genetic divergence estimated for SNP58 (PIP2), SNP37 (ERECTA) and SNP56 (PIP2) was significantly correlated, and in each case the hierarchical relationships co-varied with dissimilarity in _CLIM_PCA1 (Table 3, Figure 5). Given low linkage disequilibrium (LD) between SNP37 and SNP56 (R^2^ = 0.008) co-variation of their inferred population genetic structure could point to concerted selection acting upon unlinked loci, both in genes influencing plant water relations, in response to a common selection pressure [60]. Conversely, the correlation in divergence patterns with SNP58 and SNP56, which reside in the PIP2 gene, more likely reflects linkage over short physical distances (R^2^ = 0.41).

### Conclusions

Inference of local adaptation in *E. camaldulensis* based on these results is limited by the small number of loci and individuals examined; however they draw our attention to the potential for further studies of adaptive variation in this species, and suggest that selection in response to climate has driven genetic differences among populations at the landscape scale. With the generation of genome wide SNP datasets which partition adaptive and neutral genetic variation there arises opportunity for application of genetic markers for the management of forest resources in the face of climate change. This could include monitoring populations for evidence of, or assessing potential for, genetic adaptation by measuring standing genetic diversity and screening adaptively important variants in populations under threat [7], [15], [93], [94]. Linking SNP diversity at putatively adaptive loci with phenotypic variation via association studies achieves an important validation of adaptive variants identified in population genetic studies, and provides a tangible mechanism by which managers can assess adaptive phenotypes in natural and planted forests. In *E. camaldulensis*, interrogation of larger SNP data sets at the landscape scale, complemented by genotype-phenotype association studies under different environments should be the next steps towards generating data sets which could be applied to these ends.

## Supporting Information

Figure S1 Latitudinal clines were obseved for each of the six principal components derrived from environmental variables. Latitude (deg.) is plotted on the x-axis and PCA casewise scores for populations on the y-axis in each case.
(TIF)

Figure S2 Mantel correlation of pairwise population F_ST_ estimated on all SNP loci as compared to the 15 nuSSR loci from Butcher et al 2009 (R^2^ = 0.27, p<0.001).
(TIF)

Figure S3 Genetic relationships among populations, inferred from principal coordinate analysis (PCoA) for (**a**) 59 SNP and (**b**) 15 nuSSR markers which indicate grouping by sub species: subsp. minima (•), subsp. obtusa (o), subsp. arida (♦), subsp. refulgens (Δ), subsp. simulata (◊) and subsp. camaldulensis (▴).
(TIF)

Figure S4 Mantel correlation of pairwise population F_ST_ estimated from ANOVA variance components on all SNP loci as compared to physical distance between populations in kilometres (km) identified significant isolation by distance (R^2^ = 0.36, p<0.001).
(TIF)

Figure S5 Heterozygosity (y-axis) estimated within populations for outlier genes plotted as a function of environment (_CLIM_PCA1) for: **a**) Dehydrin (R^2^ = 0.44; p<0.001), **b**) PIP2 (R^2^ = 0.39; p<0.003), **c**) COMT (R^2^ = 0.45; p<0.001).
(TIF)

Figure S6 Variation in population level allele frequency (x-axis) for the six outlier SNP loci presented in Table 3 and principal components derived from environmental variables (y-axis) which were significantly associated.
(TIF)

Table S1 Mean annual estaimtes for environmental variables applied in principal component analyses.
(DOCX)

Table S2 Correlations (loading) between environmental variables and principal components.
(DOCX)

Table S3 Summary of the 59 SNP loci used in this study. Minor allele frequency (MAF) was determined over all populations. Amino acid abbreviations according to IUPAC conventions.
(DOCX)

Table S4 Biallelic genotype calls for all SNP loci screened across each of the 191 *E. camaldulensis* individuals used in this study.
(CSV)

Table S5 Whole genes and SNP loci identified as having divergence more extreme than expected when compared to the neutral distribution simualted in Arlequin.
(DOCX)

Table S6 SNP loci for which a model including selection was supported following analyses with BayeScan.
(DOCX)

Table S7 SNP alleles exhibiting significant covariation (p≤0.05) with environment following bonferroni correction for both the Wald and likelihood ratio test implemented in SAM.
(DOCX)

Table S8 Significant assocaitions (p≤0.05) identified with component environemntal variables following adjustment for popualtion structure and multiple testing (permutation) in Tassel.
(DOCX)
