# Supplementary figures and images for: Characterisation of Adaptive Genetic Diversity in Environmentally Contrasted Populations of Eucalyptus camaldulensis Dehnh. (River Red Gum)
Source: PLoS One. 2014 Aug 5;9(8):e103515. doi: 10.1371/journal.pone.0103515 (PMC4122390; doi:10.1371/journal.pone.0103515)

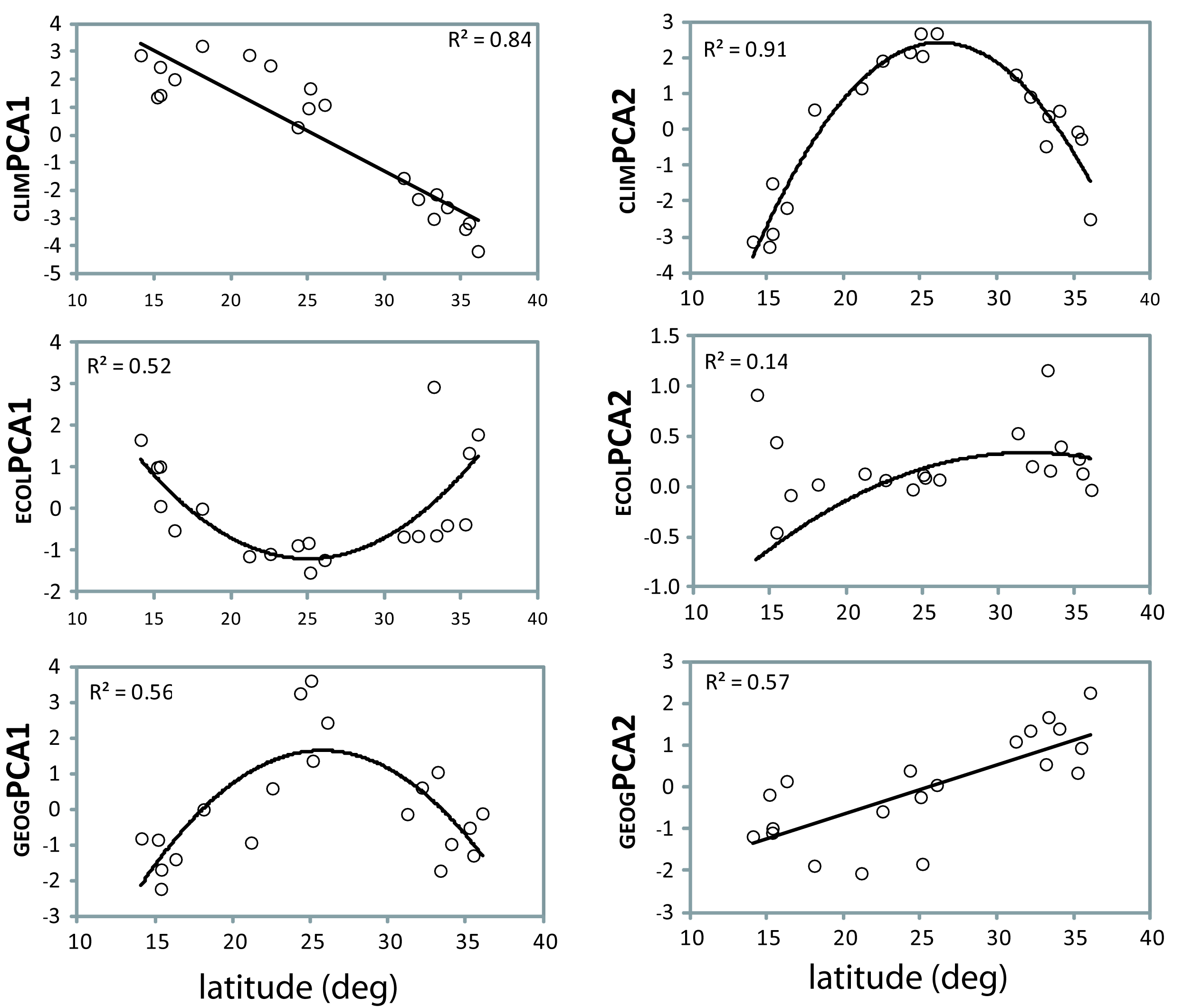

Supplement: Figure S1 — Latitudinal clines were obseved for each of the six principal components derrived from environmental variables. Latitude (deg.) is plotted on the x-axis and PCA casewise scores for populations on the y-axis in each case. (TIF) [file pone.0103515.s001.tif]

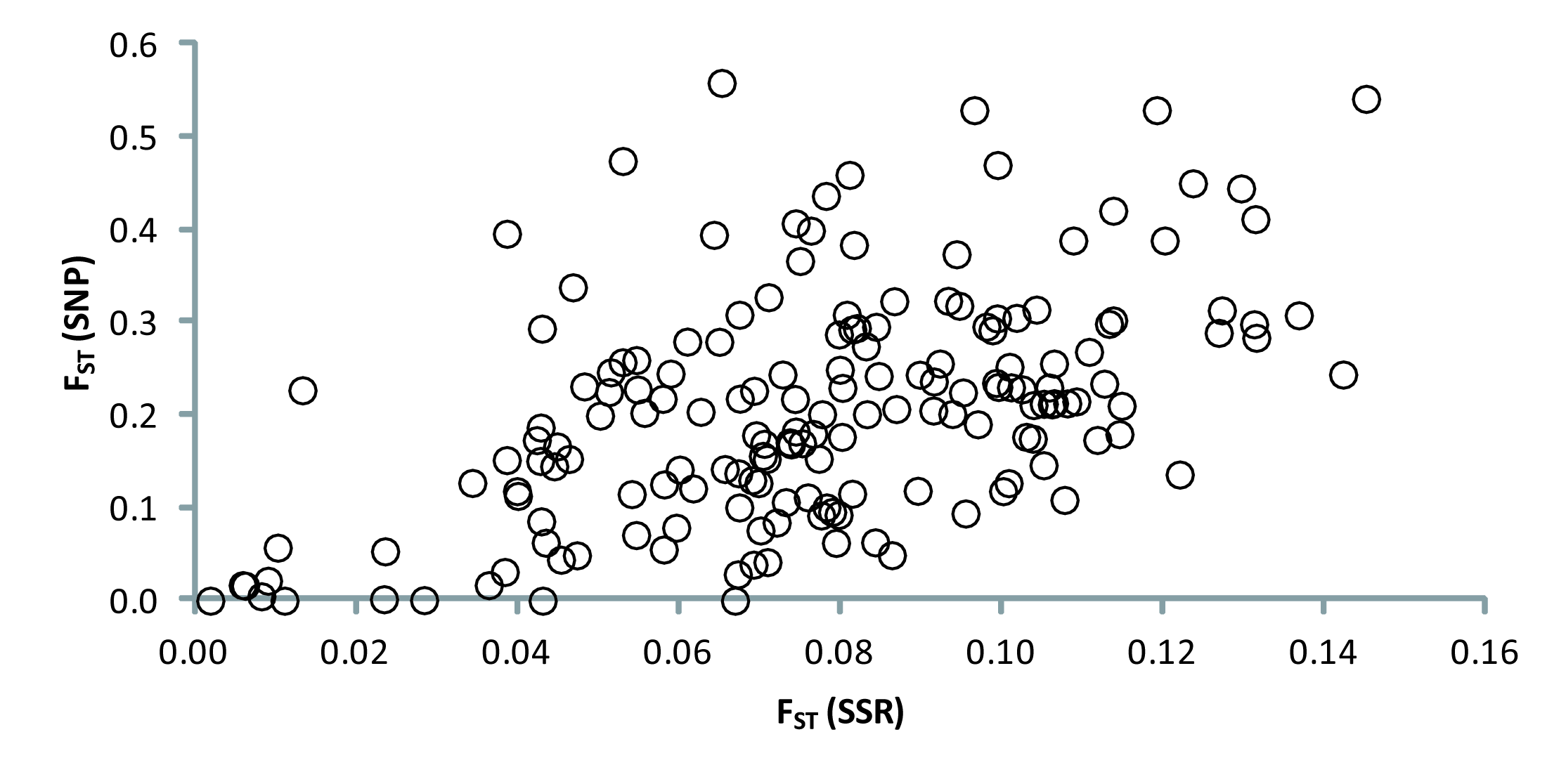

Supplement: Figure S2 — Mantel correlation of pairwise population FST estimated on all SNP loci as compared to the 15 nuSSR loci from Butcher et al 2009 (R2 = 0.27, p<0.001). (TIF) [file pone.0103515.s002.tif]

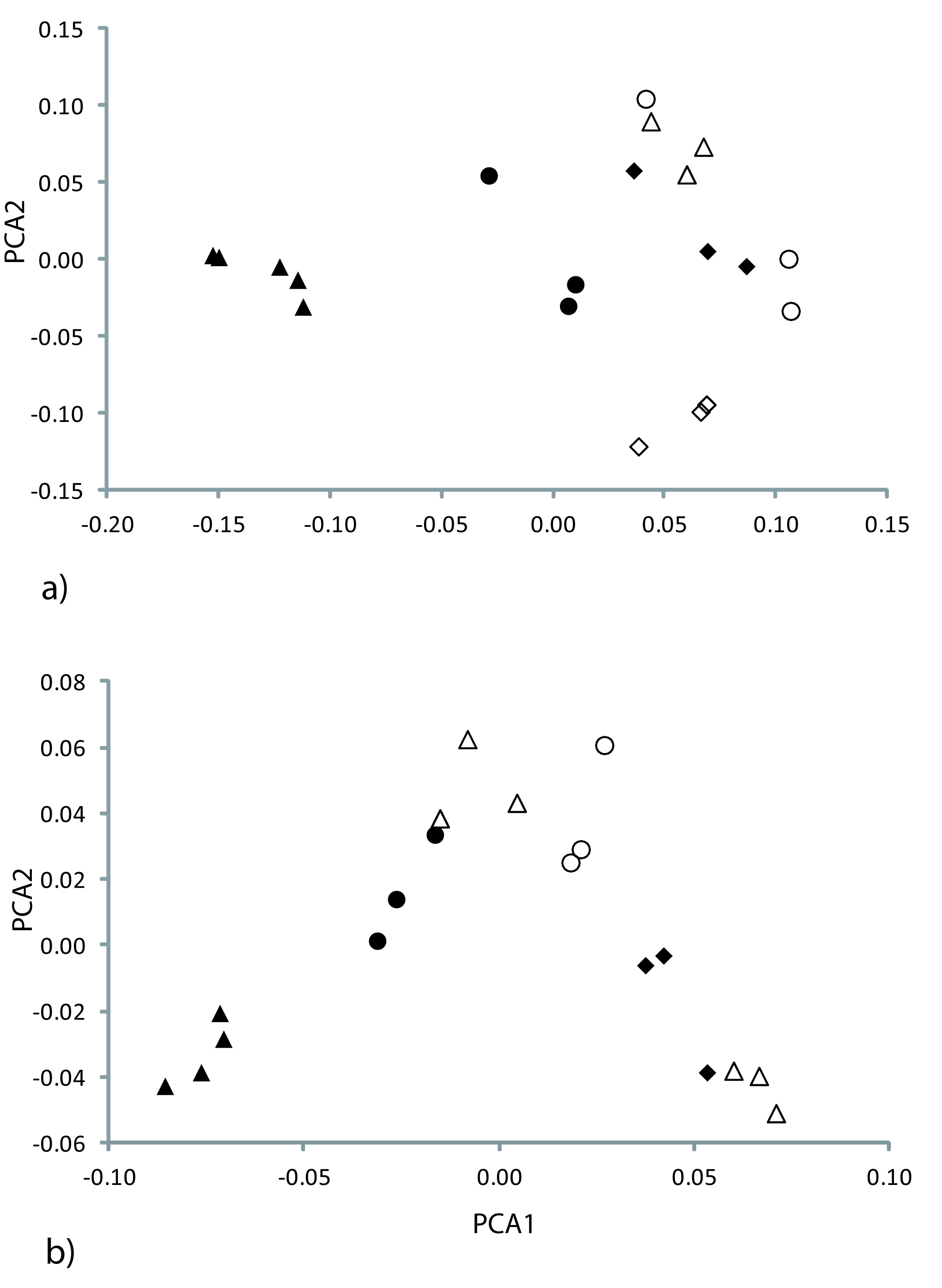

Supplement: Figure S3 — Genetic relationships among populations, inferred from principal coordinate analysis (PCoA) for (a) 59 SNP and (b) 15 nuSSR markers which indicate grouping by sub species: subsp. minima (•), subsp. obtusa (o), subsp. arida (♦), subsp. refulgens (Δ), subsp. simulata (◊) and subsp. camaldulensis (▴). (TIF) [file pone.0103515.s003.tif]

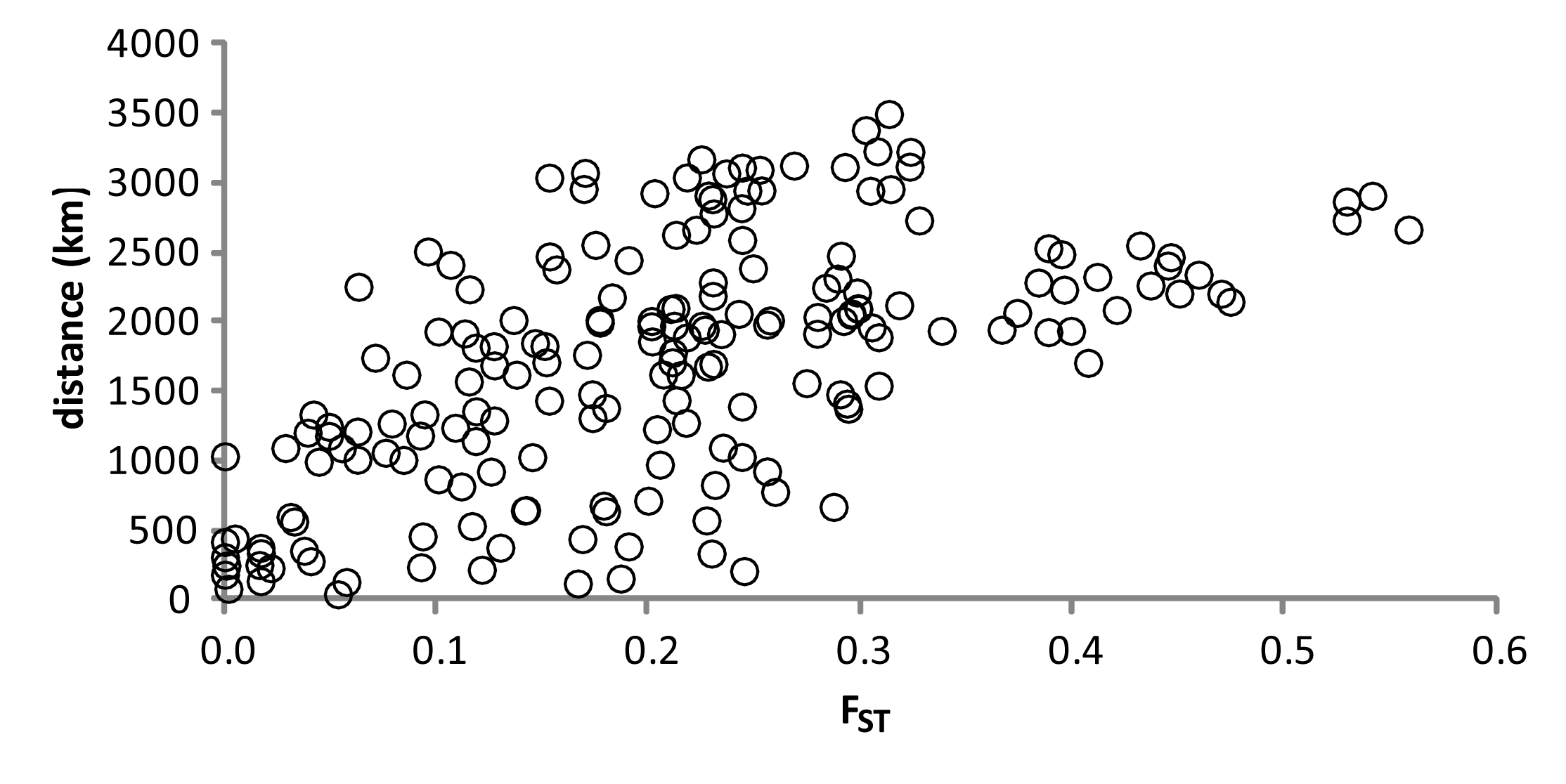

Supplement: Figure S4 — Mantel correlation of pairwise population FST estimated from ANOVA variance components on all SNP loci as compared to physical distance between populations in kilometres (km) identified significant isolation by distance (R2 = 0.36, p<0.001). (TIF) [file pone.0103515.s004.tif]

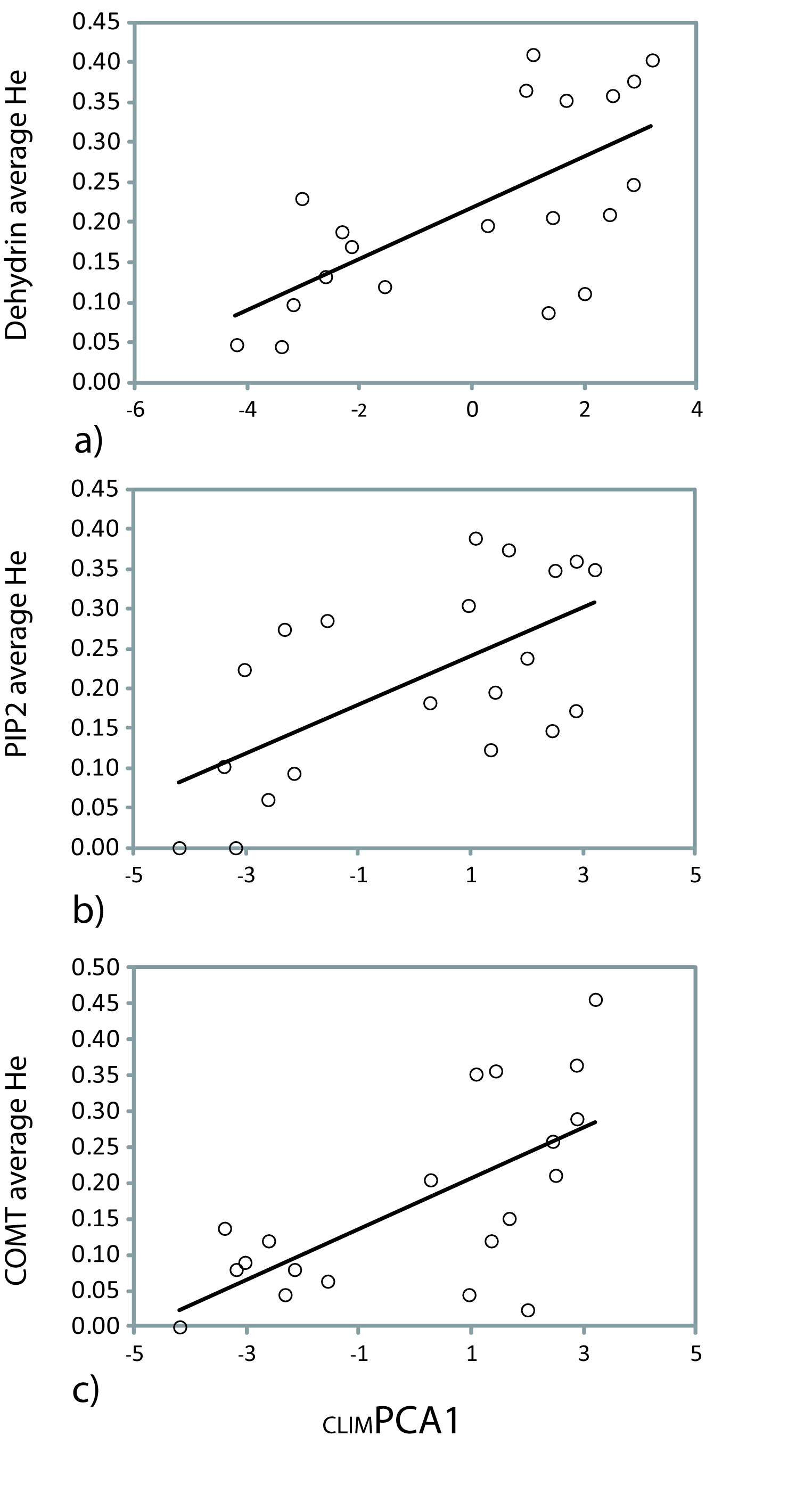

Supplement: Figure S5 — Heterozygosity (y-axis) estimated within populations for outlier genes plotted as a function of environment (CLIMPCA1) for: a) Dehydrin (R2 = 0.44; p<0.001), b) PIP2 (R2 = 0.39; p<0.003), c) COMT (R2 = 0.45; p<0.001). (TIF) [file pone.0103515.s005.tif]

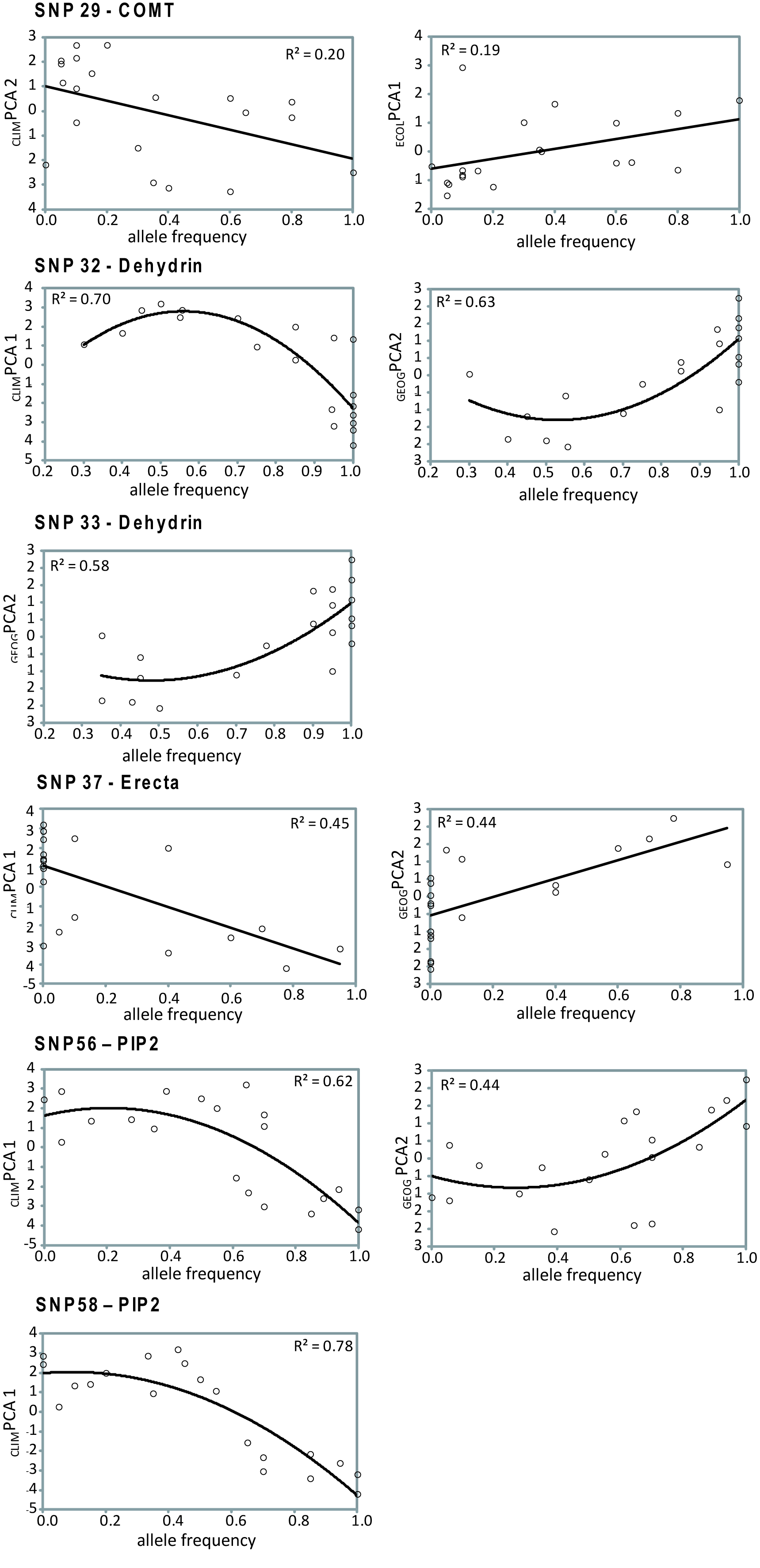

Supplement: Figure S6 — Variation in population level allele frequency (x-axis) for the six outlier SNP loci presented in Table 3 and principal components derived from environmental variables (y-axis) which were significantly associated. (TIF) [file pone.0103515.s006.tif]
