# Supplementary material for: Characterisation of Adaptive Genetic Diversity in Environmentally Contrasted Populations of Eucalyptus camaldulensis Dehnh. (River Red Gum)
Source: PLoS One. 2014 Aug 5;9(8):e103515. doi: 10.1371/journal.pone.0103515 (PMC4122390; doi:10.1371/journal.pone.0103515)
Supplement: Table S1 — Mean annual estaimtes for environmental variables applied in principal component analyses. (DOCX) [file pone.0103515.s007.docx]

|  | Fortescue | Kooline | Elong | Fitzroy Crossing | Warburton | Giles Creek | Victoria River lower | Edith River | Palmer River | Huddleston | Bunyeroo Creek | Boolcunda Creek | Wirrengren Plain | Wentworth | Mitchell River | Laura River | Normanby River | Barmah | Hillston | Towong |
| --- | --- | --- | --- | --- | --- | --- | --- | --- | --- | --- | --- | --- | --- | --- | --- | --- | --- | --- | --- | --- |
|  |  |  |  |  |  |  |  |  |  |  |  |  |  |  |  |  |  |  |  |  |
|  |  |  |  |  |  |  |  |  |  |  |  |  |  |  |  |  |  |  |  |  |
| **a) Climate** |  |  |  |  |  |  |  |  |  |  |  |  |  |  |  |  |  |  |  |  |
| precipitation (cm) | 23.3 | 24.7 | 16.9 | 43.8 | 17.2 | 20.7 | 70.3 | 87.8 | 20.2 | 38 | 19.9 | 23.5 | 25.7 | 21.9 | 78.3 | 85.2 | 101.9 | 31.7 | 28.2 | 66.5 |
| min. temperature (deg. C) | 18.7 | 18.3 | 16 | 19.1 | 14.2 | 15 | 20.1 | 19.7 | 12.8 | 9.7 | 11.2 | 9.7 | 8.6 | 9.5 | 19 | 19.6 | 18.9 | 8.9 | 10.4 | 6.7 |
| max. temperature (deg. C) | 32.9 | 33.6 | 30.8 | 35.3 | 29.3 | 28.9 | 34 | 33.7 | 29.5 | 22.7 | 24.7 | 23.3 | 22.6 | 23.9 | 32.3 | 30.6 | 28.8 | 22.1 | 24 | 20.8 |
| evaporation (mm) | 276 | 276 | 264 | 247 | 272 | 271 | 217 | 189 | 248 | 163 | 228 | 192 | 138 | 166 | 194 | 180 | 168 | 146 | 167 | 113 |
| wet bulb 9am temperature (deg. C) | 19 | 17.4 | 15.3 | 20.3 | 13 | 12 | 21 | 21.1 | 13 | 12.2 | 12.6 | 11.7 | 11.5 | 12 | 21.1 | 22 | 21.4 | 11.7 | 12.5 | 10.8 |
| wet bulb 3pm temperature (deg. C) | 21 | 19.3 | 17.6 | 21.4 | 15.9 | 15.3 | 22.6 | 22.7 | 16.2 | 14.4 | 15.1 | 14.3 | 14.4 | 14.9 | 22.8 | 23.2 | 22.4 | 14.3 | 15.2 | 13.9 |
| aridity index | 0.1 | 0.1 | 0.1 | 0.2 | 0.1 | 0.1 | 0.3 | 0.5 | 0.1 | 0.5 | 0.1 | 0.2 | 0.3 | 0.2 | 0.4 | 0.5 | 0.6 | 0.4 | 0.2 | 1 |
| moisture variability (%) | 14 | 12 | 11 | 13 | 16 | 17 | 9 | 10 | 19 | 9 | 12 | 9 | 9 | 11 | 14 | 15 | 19 | 8 | 11 | 9 |
| vapour pressure deficit (VPD) (KPa) | 1.3 | 1.7 | 1.4 | 1.6 | 1.3 | 1.4 | 1.3 | 1.2 | 1.2 | 0.4 | 0.8 | 0.6 | 0.4 | 0.6 | 0.9 | 0.6 | 0.5 | 0.4 | 0.6 | 0.3 |
| humidity, relative (%) | 61.5 | 59.1 | 61.6 | 59.7 | 59.3 | 58.2 | 68.6 | 73.4 | 62.2 | 75.6 | 67 | 71.2 | 77.2 | 74.9 | 73.7 | 79 | 80.3 | 77.5 | 73.3 | 84.5 |
| irradiance (MJ/m2/day) | 18.1 | 18.8 | 16.7 | 18.8 | 18.2 | 19.5 | 20.3 | 18.5 | 22.1 | 22.7 | 23.3 | 21.9 | 23.1 | 22.7 | 19.7 | 20.3 | 19.6 | 21.8 | 21.5 | 21.7 |
| runoff (megalitres/5x5km/year) | 1772 | 457 | 401 | 1722 | 26 | 395 | 2774 | 5398 | 343 | 107 | 247 | 316 | 10 | 0 | 2427 | 1933 | 2043 | 136 | 134 | 162 |

|  | Fortescue | Kooline | Elong | Fitzroy Crossing | Warburton | Giles Creek | Victoria River lower | Edith River | Palmer River | Huddleston | Bunyeroo Creek | Boolcunda Creek | Wirrengren Plain | Wentworth | Mitchell River | Laura River | Normanby River | Barmah | Hillston | Towong |
| --- | --- | --- | --- | --- | --- | --- | --- | --- | --- | --- | --- | --- | --- | --- | --- | --- | --- | --- | --- | --- |
|  |  |  |  |  |  |  |  |  |  |  |  |  |  |  |  |  |  |  |  |  |
|  |  |  |  |  |  |  |  |  |  |  |  |  |  |  |  |  |  |  |  |  |
| **b) Ecology** |  |  |  |  |  |  |  |  |  |  |  |  |  |  |  |  |  |  |  |  |
| endemism (endemism x 100) | 0.28 | 0.87 | 0.18 | 0.54 | 0 | 1.18 | 0.45 | 1.93 | 0.52 | 0.17 | 0.07 | 0.24 | 0.06 | 0.15 | 0 | 3.12 | 0.45 | 0.13 | 0.05 | 1.47 |
| species richness (freq.) | 0.4 | 0.4 | 0 | 0.9 | 0.1 | 0.9 | 2.9 | 5.4 | 0.1 | 6.6 | 2 | 1 | 1.4 | 1.8 | 0 | 0 | 0.7 | 2 | 0.8 | 2.1 |
| NDVI (NDVI x 100) | 21 | 22 | 16 | 42 | 22 | 24 | 47 | 40 | 29 | 59 | 18 | 27 | 30 | 26 | 38 | 49 | 48 | 62 | 29 | 70 |

|  | Fortescue | Kooline | Elong | Fitzroy Crossing | Warburton | Giles Creek | Victoria River lower | Edith River | Palmer River | Huddleston | Bunyeroo Creek | Boolcunda Creek | Wirrengren Plain | Wentworth | Mitchell River | Laura River | Normanby River | Barmah | Hillston | Towong |
| --- | --- | --- | --- | --- | --- | --- | --- | --- | --- | --- | --- | --- | --- | --- | --- | --- | --- | --- | --- | --- |
|  |  |  |  |  |  |  |  |  |  |  |  |  |  |  |  |  |  |  |  |  |
|  |  |  |  |  |  |  |  |  |  |  |  |  |  |  |  |  |  |  |  |  |
| **c) Geography** |  |  |  |  |  |  |  |  |  |  |  |  |  |  |  |  |  |  |  |  |
| latitude (deg.) | 21.2 | 22.6 | 25.2 | 18.1 | 26.1 | 25 | 15.4 | 14.1 | 24.3 | 33.2 | 31.2 | 32.2 | 35.3 | 34.1 | 16.3 | 15.4 | 15.2 | 35.5 | 33.4 | 36.1 |
| longitude (deg.) | 116 | 116 | 116 | 125 | 126 | 128 | 130 | 132 | 132 | 138 | 138 | 138 | 142 | 142 | 143 | 144 | 145 | 145 | 145 | 148 |
| aspect (deg.) | 102 | 298 | 21 | 42 | 200 | 103 | 250 | 182 | 191 | 114 | 259 | 280 | 46 | 269 | 288 | 98 | 207 | 152 | 331 | 313 |
| elevation (m) | 25 | 223 | 352 | 165 | 476 | 618 | 2 | 154 | 613 | 424 | 70 | 257 | 59 | 43 | 128 | 90 | 73 | 97 | 109 | 259 |
| soil depth (m) | 1.1 | 0.6 | 1 | 0.8 | 0.5 | 0.4 | 1.4 | 1 | 0.2 | 0.7 | 0.7 | 0.8 | 0.7 | 0.7 | 1.1 | 1.3 | 1 | 1.2 | 1.3 | 0.9 |
