# Supplementary material for: Characterisation of Adaptive Genetic Diversity in Environmentally Contrasted Populations of Eucalyptus camaldulensis Dehnh. (River Red Gum)
Source: PLoS One. 2014 Aug 5;9(8):e103515. doi: 10.1371/journal.pone.0103515 (PMC4122390; doi:10.1371/journal.pone.0103515)
Supplement: Table S2 — Correlations (loading) between environmental variables and principal components. (DOCX) [file pone.0103515.s008.docx]

| **a) Climate** |  |  |
| --- | --- | --- |
| Variable | _CLIM_PCA1 | _CLIM_PCA2 |
| rain. | 0.290 | **-0.933** |
| min. T | **0.963** | -0.224 |
| max. T | **0.985** | -0.010 |
| evap. | **0.712** | **0.678** |
| wet bulb 9am | **0.819** | -0.527 |
| wet bulb 3pm | **0.840** | -0.507 |
| aridity index | -0.309 | **-0.845** |
| moisture variability | 0.486 | 0.164 |
| VPD | **0.817** | 0.496 |
| humidity | -0.578 | **-0.800** |
| irradiance | **-0.821** | -0.079 |
| runoff | 0.648 | **-0.620** |

| **b) Ecology** | |  |
| --- | --- | --- |
| Variable | _ECOL_PCA1 | _ECOL_PCA2 |
| endemism | 0.214 | **-0.944** |
| species Richness | **0.809** | 0.403 |
| NDVI Mean | **0.880** | -0.140 |

| **c) Geography** | |  |
| --- | --- | --- |
| Variable | _GEOG_PCA1 | _GEOG_PCA2 |
| latitude | 0.157 | **0.754** |
| longitude | -0.406 | **0.727** |
| aspect | -0.242 | **0.635** |
| elevation | **0.941** | 0.061 |
| soil depth | **-0.861** | -0.175 |

Component loadings highlighted in bold where exceeded ± 0.5, indicating variable was a major contirbutor to the principal component in question
