# Supplementary material for: Characterisation of Adaptive Genetic Diversity in Environmentally Contrasted Populations of Eucalyptus camaldulensis Dehnh. (River Red Gum)
Source: PLoS One. 2014 Aug 5;9(8):e103515. doi: 10.1371/journal.pone.0103515 (PMC4122390; doi:10.1371/journal.pone.0103515)
Supplement: Table S3 — Summary of the 59 SNP loci used in this study. Minor allele frequency (MAF) was determined over all populations. Amino acid abbreviations according to IUPAC conventions. (DOCX) [file pone.0103515.s009.docx]

| **locus** | **gene** | **SNP** | **MAF** | **SNP type** | **amino acid** | **gene region** |
| --- | --- | --- | --- | --- | --- | --- |
| SNP1 | CAD | A/G | 0.47 | silent | - | intron |
| SNP2 | CAD | C/T | 0.42 | silent | - | intron |
| SNP3 | CAD | C/T | 0.05 | silent | - | intron |
| SNP4 | CAD | A/G | 0.32 | synonymous | Ser | exon |
| SNP5 | CAD | A/C | 0.03 | synonymous | Arg | exon |
| SNP6 | CCR | C/T | 0.11 | synonymous | Ser | exon |
| SNP7 | CCR | A/G | 0.17 | synonymous | Ala | exon |
| SNP8 | CCR | A/G | 0.05 | non-synonymous | Ile/Val | exon |
| SNP9 | CCR | C/G | 0.35 | silent | - | intron |
| SNP10 | CesA1 | C/T | 0.49 | silent | - | intron |
| SNP11 | CesA1 | C/T | 0.11 | synonymous | Val | exon |
| SNP12 | CesA1 | C/T | 0.08 | synonymous | Ile | exon |
| SNP13 | CesA1 | A/G | 0.33 | synonymous | Leu | exon |
| SNP14 | CesA1 | A/G | 0.27 | synonymous | Ser | exon |
| SNP15 | CesA1 | C/T | 0.25 | synonymous | Ser | exon |
| SNP16 | CesA3 | C/T | 0.03 | silent | - | 5'UTR |
| SNP17 | CesA3 | C/T | 0.17 | synonymous | His | exon |
| SNP18 | CesA3 | A/G | 0.06 | synonymous | Pro | exon |
| SNP19 | CesA3 | C/T | 0.03 | synonymous | Val | exon |
| SNP20 | CesA3 | C/T | 0.48 | synonymous | Gly | exon |
| SNP21 | COBL4 | A/T | 0.22 | silent | - | intron |
| SNP22 | COBL4 | C/T | 0.03 | silent | - | Intron |
| SNP23 | COBL4 | C/T | 0.06 | silent | - | intron |
| SNP24 | COBL4 | C/T | 0.06 | synonymous | Leu | exon |
| SNP25 | COBL4 | C/T | 0.18 | silent | - | intron |
| SNP26 | COBL4 | A/T | 0.18 | non-synonymous | Val/Glu | exon |
| SNP27 | COBL4 | A/T | 0.02 | synonymous | Gly | exon |
| SNP28 | COMT | C/T | 0.02 | silent | - | intron |
| SNP29 | COMT | A/G | 0.48 | silent | - | intron |
| SNP30 | COMT | C/T | 0.1 | silent | - | intron |
| SNP31 | COMT | A/T | 0.07 | silent | - | intron |
| SNP32 | Dehydrin | A/G | 0.14 | synonymous | Arg | exon |
| SNP33 | Dehydrin | G/T | 0.14 | synonymous | Val | exon |
| SNP34 | Dehydrin | A/G | 0.32 | synonymous | Ala | exon |
| SNP35 | Dehydrin | A/G | 0.08 | synonymous | Thr | exon |
| SNP36 | ERECTA | C/T | 0.35 | - | - | - |
| SNP37 | ERECTA | G/T | 0.41 | silent | - | intron |
| SNP38 | ERECTA | A/C | 0.36 | - | - | - |
| SNP39 | ERECTA | C/T | 0.46 | - | - | - |
| SNP40 | Korrigan | A/T | 0.03 | non-synonymous | Thr/Ala | exon |
| SNP41 | Korrigan | A/C | 0.29 | non-synonymous | Asp/Ala | exon |
| SNP42 | Korrigan | A/G | 0.26 | synonymous | Ser | exon |

| **locus** | **gene** | **SNP** | **MAF** | **SNP type** | **amino acid** | **gene region** |
| --- | --- | --- | --- | --- | --- | --- |
| SNP43 | Korrigan | C/T | 0.29 | synonymous | Thr | exon |
| SNP44 | Korrigan | C/T | 0.26 | synonymous | Ile | exon |
| SNP45 | Korrigan | A/G | 0.43 | non-synonymous | His/Arg | exon |
| SNP46 | Korrigan | C/T | 0.12 | synonymous | Asp | exon |
| SNP47 | MYB4 | A/G | 0.04 | non-synonymous | Met/Val | exon |
| SNP48 | MYB4 | C/G | 0.1 | silent | - | intron |
| SNP49 | MYB4 | A/C | 0.02 | silent | - | intron |
| SNP50 | MYB4 | A/G | 0.05 | non-synonymous | Ala/Thr | exon |
| SNP51 | bZIP | C/G | 0.09 | - | - | - |
| SNP52 | bZIP | C/T | 0.46 | - | - | - |
| SNP53 | bZIP | C/G | 0.05 | silent | - | 5'UTR |
| SNP54 | bZIP | C/T | 0.31 | - | - | - |
| SNP55 | PIP2 | C/T | 0.26 | silent | - | intron |
| SNP56 | PIP2 | C/T | 0.32 | synonymous | Leu | exon |
| SNP57 | PIP2 | C/T | 0.03 | synonymous | Ala | exon |
| SNP58 | PIP2 | C/T | 0.36 | silent | - | intron |
| SNP59 | PIP2 | A/G | 0.04 | silent | - | intron |
