# Supplementary material for: Characterisation of Adaptive Genetic Diversity in Environmentally Contrasted Populations of Eucalyptus camaldulensis Dehnh. (River Red Gum)
Source: PLoS One. 2014 Aug 5;9(8):e103515. doi: 10.1371/journal.pone.0103515 (PMC4122390; doi:10.1371/journal.pone.0103515)
Supplement: Table S5 — Whole genes and SNP loci identified as having divergence more extreme than expected when compared to the neutral distribution simualted in Arlequin. (DOCX) [file pone.0103515.s011.docx]

| **locus** | **gene** | **SNP** | **SNP type** | **gene region** | **F_ST_** | **p-val** | **q-value** |
| --- | --- | --- | --- | --- | --- | --- | --- |
| whole gene | PIP2 | - | - | - | 0.4 | 0.002 | 0.010 |
| whole gene | COMT | - | - | - | 0.29 | 0.007 | 0.017 |
| whole gene | Dehydrin | - | - | - | 0.32 | 0.010 | 0.020 |
| whole gene | ERECTA | - | - | - | 0.24 | 0.036 | 0.045 |
| SNP57 | PIP2 | C/T | synonymous | exon | 0.41 | 0.000 | 0.000 |
| SNP37 | ERECTA | G/T | silent | intron | 0.45 | 0.000 | 0.000 |
| SNP58 | PIP2 | C/T | silent | intron | 0.45 | 0.001 | 0.000 |
| SNP33 | Dehydrin like | G/T | synonymous | exon | 0.39 | 0.002 | 1.00E-06 |
| SNP55 | PIP2 | C/T | silent | intron | 0.44 | 0.002 | 1.00E-06 |
| SNP29 | COMT | A/G | silent | intron | 0.39 | 0.003 | 1.00E-06 |
| SNP56 | PIP2 | C/T | synonymous | exon | 0.35 | 0.006 | 2.00E-06 |
| SNP32 | Dehydrin like | A/G | synonymous | exon | 0.34 | 0.007 | 1.80E-05 |
| SNP34 | Dehydrin like | A/G | synonymous | exon | 0.37 | 0.007 | 4.90E-05 |
| SNP25 | COBL4 | C/T | silent | intron | 0.34 | 0.012 | 0.003 |
| SNP30 | COMT | C/T | silent | intron | 0.26 | 0.032 | 0.006 |
| SNP8 | CCR | A/G | non-synonymous | exon | 0.25 | 0.025 | 0.008 |
| SNP53 | bZIP | C/G | silent | 5'UTR | 0.22 | 0.030 | 0.042 |
