# Supplementary material for: Characterisation of Adaptive Genetic Diversity in Environmentally Contrasted Populations of Eucalyptus camaldulensis Dehnh. (River Red Gum)
Source: PLoS One. 2014 Aug 5;9(8):e103515. doi: 10.1371/journal.pone.0103515 (PMC4122390; doi:10.1371/journal.pone.0103515)
Supplement: Table S6 — SNP loci for which a model including selection was supported following analyses with BayeScan. (DOCX) [file pone.0103515.s012.docx]

| **locus** | **gene** | **SNP** | **SNP type** | **gene region** | **F_ST_** | **Log_10_(odds)** | **alpha** | **evidence of selection** | **selection type** |
| --- | --- | --- | --- | --- | --- | --- | --- | --- | --- |
| SNP32 | Dehydrin | A/G | synonymous | exon | 0.30 | 0.26 | 0.59 | weak | diversifying |
| SNP33 | Dehydrin | G/T | synonymous | exon | 0.30 | 0.33 | 0.6 | weak | diversifying |
| SNP37 | ERECTA | G/T | silent | intron | 0.42 | 1.74 | 1.2 | very strong | diversifying |
| SNP56 | Pip2 | C/T | synonymous | exon | 0.31 | 0.54 | 0.66 | substantial | diversifying |
| SNP58 | Pip2 | C/T | silent | intron | 0.37 | 1.78 | 0.98 | very strong | diversifying |

Log_10_(odds) = logarithm of Posterior Odds to base 10 for the model including selection

alpha = strength and direction of selection. A positive value of alpha suggests diversifying selection

Evidence of selection was inferred from posterior odds using Jeffrey’s scale (Foll and Gaggiotti 2008)
