# Supplementary material for: Characterisation of Adaptive Genetic Diversity in Environmentally Contrasted Populations of Eucalyptus camaldulensis Dehnh. (River Red Gum)
Source: PLoS One. 2014 Aug 5;9(8):e103515. doi: 10.1371/journal.pone.0103515 (PMC4122390; doi:10.1371/journal.pone.0103515)
Supplement: Table S7 — SNP alleles exhibiting significant covariation (p≤0.05) with environment following bonferroni correction for both the Wald and likelihood ratio test implemented in SAM. (DOCX) [file pone.0103515.s013.docx]

| **locus** | **allele** | **gene** | **_CLIM_PCA1** | **_CLIM_PCA2** | **_ECOL_PCA1** | **_ECOL_PCA2** | **_GEOG_PCA1** | **_GEOG_PCA2** |
| --- | --- | --- | --- | --- | --- | --- | --- | --- |
| SNP13 | G | CesA1 | + |  |  |  |  |  |
| SNP21 | T | COBL4 |  |  |  |  |  | + |
| SNP25 | C | COBL4 | + |  |  |  |  |  |
| SNP26 | A | COBL4 | + |  |  |  |  | + |
| SNP29 | A | COMT | + | + | + |  |  |  |
| SNP29 | G | COMT | + |  | + |  |  | + |
| SNP30 | T | COMT | + |  |  |  |  | + |
| SNP32 | G | Dehydrin like | + |  |  |  |  | + |
| SNP33 | T | Dehydrin like | + |  |  |  |  | + |
| SNP34 | G | Dehydrin like | + |  |  |  |  | + |
| SNP37 | G | ERECTA | + |  |  |  |  | + |
| SNP37 | T | ERECTA | + |  |  |  |  | + |
| SNP38 | C | ERECTA | + |  |  |  |  |  |
| SNP39 | C | ERECTA | + |  |  |  |  |  |
| SNP39 | T | ERECTA | + |  |  |  |  |  |
| SNP40 | A | Korrigan |  |  |  | + |  |  |
| SNP45 | G | Korrigan | + |  | + |  |  | + |
| SNP46 | T | Korrigan | + |  |  |  |  | + |
| SNP50 | A | MYB4 |  |  |  |  |  | + |
| SNP54 | T | bZIP | + |  |  |  |  |  |
| **SNP55** | C | PIP2 | + |  |  |  |  | + |
| **SNP55** | T | PIP2 | + |  |  |  |  |  |
| **SNP56** | C | PIP2 | + |  |  |  |  |  |

| **marker** | **allele** | **gene** | **_CLIM_PCA1** | **_CLIM_PCA2** | **_ECOL_PCA1** | **_ECOL_PCA2** | **_GEOG_PCA1** | **_GEOG_PCA2** |
| --- | --- | --- | --- | --- | --- | --- | --- | --- |
| SNP56 | T | PIP2 | + |  |  |  |  | + |
| SNP58 | C | PIP2 | + |  |  |  |  | + |
| SNP58 | T | PIP2 | + |  |  |  |  | + |
| SNP59 | G | PIP2 |  |  |  |  |  | + |
| SNP7 | A | CCR |  | + |  |  |  |  |
| SNP8 | A | CCR |  |  |  |  |  | + |
