# Supplementary material for: Characterisation of Adaptive Genetic Diversity in Environmentally Contrasted Populations of Eucalyptus camaldulensis Dehnh. (River Red Gum)
Source: PLoS One. 2014 Aug 5;9(8):e103515. doi: 10.1371/journal.pone.0103515 (PMC4122390; doi:10.1371/journal.pone.0103515)
Supplement: Table S8 — Significant assocaitions (p≤0.05) identified with component environemntal variables following adjustment for popualtion structure and multiple testing (permutation) in Tassel. (DOCX) [file pone.0103515.s014.docx]

| **locus** | **gene** | **environment** | **p-val** | **p-val^permuted^** | **R^2^** |
| --- | --- | --- | --- | --- | --- |
| 5 | CAD | _CLIM_PCA2 | 3.4E-04 | 0.035 | 0.057 |
| 5 | CAD | _ECOL_PCA1 | 7.0E-04 | 0.023 | 0.063 |
| 7 | CCR | _CLIM_PCA2 | 2.1E-06 | 4.0E-04 | 0.087 |
| 7 | CCR | _ECOL_PCA1 | 6.0E-06 | 2.0E-04 | 0.099 |
| 10 | CesA1 | _CLIM_PCA2 | 0.001 | 0.049 | 0.054 |
| 11 | CesA1 | _ECOL_PCA2 | 1.7E-07 | 0.004 | 0.105 |
| 19 | CesA3 | _CLIM_PCA2 | 0.001 | 0.053 | 0.053 |
| 21 | COBL4 | _CLIM_PCA1 | 1.6E-04 | 0.009 | 0.045 |
| 26 | COBL4 | _CLIM_PCA1 | 3.7E-04 | 0.019 | 0.039 |
| 29 | COMT | _CLIM_PCA2 | 1.0E-07 | 0.002 | 0.074 |
| 29 | COMT | _ECOL_PCA1 | 6.0E-05 | 2.0E-04 | 0.122 |
| 30 | COMT | _CLIM_PCA1 | 3.8E-05 | 0.003 | 0.043 |
| 32 | Dehydrin-like | _CLIM_PCA1 | 6.4E-07 | 0.001 | 0.061 |
| 32 | Dehydrin-like | _GEOG_PCA2 | 4.8E-06 | 2.0E-04 | 0.088 |
| 33 | Dehydrin-like | _GEOG_PCA2 | 4.1E-04 | 0.019 | 0.043 |
| 36 | ERECTA | _CLIM_PCA1 | 3.1E-04 | 0.016 | 0.041 |
| 37 | ERECTA | _CLIM_PCA1 | 5.2E-04 | 0.024 | 0.033 |
| 37 | ERECTA | _GEOG_PCA2 | 0.001 | 0.055 | 0.039 |
| 40 | Korrigan | _ECOL_PCA2 | 4.1E-05 | 0.053 | 0.075 |
| 50 | MYB4 | _CLIM_PCA1 | 4.4E-04 | 0.044 | 0.036 |
| 50 | MYB4 | _CLIM_PCA2 | 9.5E-04 | 0.049 | 0.054 |
| 50 | MYB4 | _GEOG_PCA2 | 9.9E-04 | 0.020 | 0.049 |
| 58 | PIP2 | _CLIM_PCA1 | 2.6E-04 | 0.015 | 0.041 |
| 59 | PIP2 | _CLIM_PCA2 | 6.3E-04 | 0.046 | 0.055 |
| 59 | PIP2 | _GEOG_PCA2 | 9.3E-04 | 0.030 | 0.045 |

R^2^ = proportion of environmental variance explained by SNP in Tassel
